# Gas-solid reaction based over one-micrometer thick stable perovskite films for efficient solar cells and modules

**DOI:** 10.1038/s41467-018-06317-8

**Published:** 2018-09-24

**Authors:** Zonghao Liu, Longbin Qiu, Emilio J. Juarez-Perez, Zafer Hawash, Taehoon Kim, Yan Jiang, Zhifang Wu, Sonia R. Raga, Luis K. Ono, Shengzhong (Frank) Liu, Yabing Qi

**Affiliations:** 1 Energy Materials and Surface Sciences Unit (EMSSU), Okinawa Institute of Science and Technology Graduate University (OIST), 1919-1 Tancha, Onna-son, Kunigami-gun, Okinawa 904-0495 Japan; 2 Key Laboratory of Applied Surface and Colloid Chemistry, Ministry of Education, Shaanxi Key Laboratory for Advanced Energy Devices, Shaanxi Engineering Lab for Advanced Energy Technology, School of Materials Science and Engineering, Shaanxi Normal University, 710119 Xi’an, China; 3 Dalian National Laboratory for Clean Energy, iChEM, Dalian Institute of Chemical Physics, Chinese Academy of Sciences, 457 Zhongshan Road, 116023 Dalian, China

## Abstract

Besides high efficiency, the stability and reproducibility of perovskite solar cells (PSCs) are also key for their commercialization. Herein, we report a simple perovskite formation method to fabricate perovskite films with thickness over 1 μm in ambient condition on the basis of the fast gas−solid reaction of chlorine-incorporated hydrogen lead triiodide and methylamine gas. The resultant thick and smooth chlorine-incorporated perovskite films exhibit full coverage, improved crystallinity, low surface roughness and low thickness variation. The resultant PSCs achieve an average power conversion efficiency of 19.1 ± 0.4% with good reproducibility. Meanwhile, this method enables an active area efficiency of 15.3% for 5 cm × 5 cm solar modules. The un-encapsulated PSCs exhibit an excellent T_80_ lifetime exceeding 1600 h under continuous operation conditions in dry nitrogen environment.

## Abstract (web summary)

Perovskite solar cells often suffer from poor uniformity and reproducibility especially in case of large area devices. Here Liu et al. developed a gas−solid reaction method that enables facile fabrication of over 1 µm thick perovskite films for solar modules with high efficiency, stability and reproducibility.

## Introduction

Organic−inorganic lead halide perovskite solar cells (PSCs) have drawn a great deal of attention in the photovoltaic research community due to their high efficiency and simple manufacturing process^1–5^. The power conversion efficiency (PCE) of PSCs has reached over 22% ^6^. Similar to other kinds of thin film photovoltaics, film qualities of perovskite layers can heavily influence device performance of PSCs. Thus, a delicate control of film quality of perovskite layers is key to achieving both superior performance and high reproducibility. Many solution processable perovskite formation methods have been developed to fabricate high-quality perovskite films, such as one-step method^2,7–9^ and two-step method^10^. These methods usually involve a transformation of 2D lead halide inorganic framework to the 3D perovskite structure. This structure transformation is often accompanied with the formation of morphological defects and crystallographic structure dislocation within the grain and at grain boundaries, where defects are present as trap states causing serious charge recombination and limiting the charge carrier diffusion length (less than 1 μm)^11,12^. To ensure efficient charge collection in the presence of defects, perovskite films are usually made sufficiently thin. So far, high efficiency PSCs mainly adopt perovskite films with thickness ranging from 400 to 800 nm (Supplementary Table 1). The low thickness fluctuation tolerance and morphological defects of thin perovskite films decrease reproducibility of the fabrication process. Moreover, thin films are prone to thickness variations and pinhole defects, which increase significantly with the area.

It is obvious that thicker perovskite films are likely to reduce the risk of forming voids and pinholes due to the larger thickness fluctuation tolerance. A high-quality thick perovskite film is desirable to achieve a high device yield and reproducibility in making large-area solar modules. Besides, thicker perovskite films can not only improve the light harvesting^13,14^, but also broaden light response region by utilizing the below-band absorption^15^. As such, it is advantageous to construct a high-quality thick perovskite film with enough carrier diffusion length in PSCs to endorse both higher efficiency and manufacturing viability. However, thick perovskite films over 1 μm have been found to be generally less efficient than thin-films devices due to the poor mobility, limited carrier diffusion length and are seldom explored (Supplementary Table 1). Moreover, conventional perovskite formation methods are usually not suitable for fabricating large-area high-quality thick perovskite films. Thus, improving the quality of thick perovskite films is critical to balance the thickness and efficiency. Also, the development of new perovskite formation techniques is desirable to construct high-quality and thick perovskite films.

Recently, a perovskite formation process based on methylamine (CH_3_NH_2_) gas−solid reaction has been developed to prepare high-quality CH_3_NH_3_PbX_3_ (X = I, Br, Cl), mixed halides CH_3_NH_3_^+^-based perovskite and Cesium/CH_3_NH_3_^+^ mixed cations perovskite films^16–18^. This gas−solid reaction-based method introduces a liquid intermediate, which leads to induced morphology reconstruction, defects healing^19,20^. Meanwhile, CH_3_NH_2_ gas treatment can help reduce the defect density in perovskite films^19,20^ and also elimination of I_2_^13^. Besides, CH_3_NH_2_-induced δ-NH_2_CH=NH_2_PbI_3_ in (CH_3_NH_3_)_*x*_(NH_2_CH=NH_2_)_1−*x*_PbI_3_ is also reported to passivate trap states to improve device performance^21^. Moreover, excess solvent is absent during this perovskite formation process, which avoids the possible detrimental effect-related residue solvent. Therefore, CH_3_NH_2_ gas-based processes are promising for depositing high-quality perovskite films across large area for PSC device fabrication^22,23^.

In terms of the synthetic techniques developed for perovskite, using chloride containing raw materials to prepare perovskite is a commonly used method^3^. Although the chlorine content in resultant perovskite films is usually much lower than the nominal ratio of chlorine added in the precursor solution and in some cases the chorine content in fully annealed perovskite films is below detection limit, both the optoelectronic properties and morphology can be tuned by appropriate chlorine incorporation^24,25^. The resultant carrier diffusion length improvement^11,12^, defects density reduction^26^, optimization of film growth^27–29^, and charge tranport^30,31^ make this method effective in obtaining high-quality perovskite films. In particular, CH_3_NH_3_PbI_3−*x*_Cl_*x*_ (i.e., CH_3_NH_3_PbI_3_ incorporated with a small amount of Cl) has been reported to exhibit a longer carrier diffusion length over 1 μm compared with pure CH_3_NH_3_PbI_3_, and as a result device performance improved dramatically^11,12^.

These earlier works have inspired us to combine the CH_3_NH_2_ gas-based perovskite formation method and partial substitution of iodine ions by chorine ions to fabricate high quality over 1-μm-thick perovskite films with a sufficient charge diffusion length, which is not only beneficial for better light absorbing but also desirable for fabricating large-area solar modules with high yield and reproducibility via low-cost printing techniques^13,32^.

Here we report a simple perovskite formation method to fabricate over 1-μm-thick perovskite films based on the fast gas−solid reaction of chlorine-incorporated hydrogen lead triiodide (HPbI_3_(Cl)) and CH_3_NH_2_ gas. The comprehensive characterization results reveal that with the synergistic effect of CH_3_NH_2_ gas and partial substitution of iodine ions by chorine ions, the resultant CH_3_NH_3_PbI_3_(Cl) (MAPbI_3_(Cl)) films with a thickness over 1 μm and low thickness variation exhibit excellent film quality. The resultant PSCs gave an average PCE of 19.1% and low PCE standard deviation (±0.4%), which indicates the excellent reproducibility of this method. Meanwhile, this method enables an active area PCE of 15.3% for 5 cm × 5 cm solar modules. Besides, the un-encapsulated PSCs exhibit an excellent T_80_ lifetime exceeding 1600 h under continuous operation conditions in dry N_2_ environment. Our film stability study also offers the in-depth understanding for the underlying mechanisms responsible for device stability improvement.

## Results

### Perovskite formation

**Fig. 1:**
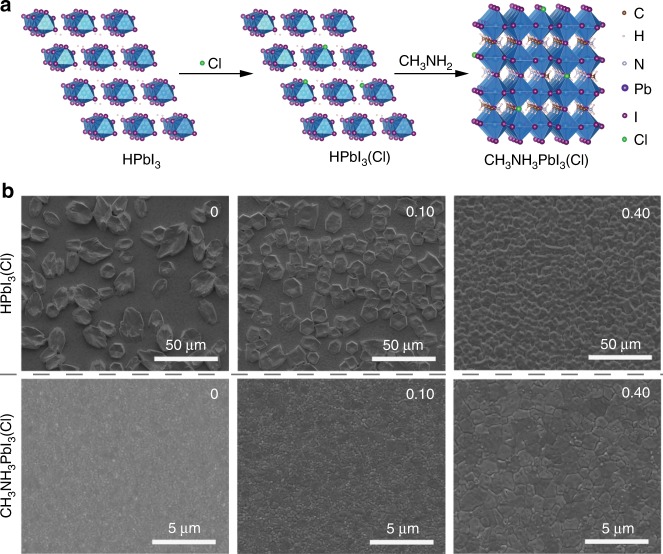
Perovskite film formation. **a** Schematic drawing showing the reaction process of partial substitution of iodine ions by chorine ions to form chlorine-incorporated perovskite MAPbI_3_(Cl) films: First, iodine ions in HPbI_3_ are partially substituted by chorine ions to form HPbI_3_(Cl). Then HPbI_3_(Cl) reacts with CH_3_NH_2_ gas to form MAPbI_3_(Cl) via the ultra-fast acid-base reaction. **b** Top-view SEM images of HPbI_3_(Cl) (top set, scale bar: 50 μm) and MAPbI_3_(Cl) (bottom set, scale bars: 5 μm) films prepared using different molar ratios of MACl versus HPbI_3_ in the HPbI_3_/MACl precursor solution

Conventional perovskite formation methods are usually not suitable for fabricating high-quality thick perovskite thin films (Supplementary Fig. 1). Here, we developed a fast gas−solid reaction of HPbI_3_(Cl) and CH_3_NH_2_ gas to prepare high-quality thick perovskite films. In this method, hydrogen lead triiodide (HPbI_3_) was used as the starting material aiming at taking advantage of its fully coordinated [PbI_3_]^−^ structure to reduce iodide-vacancy and improve film quality (Supplementary Figs. 2, 3)^33,34^. Firstly, we tuned the thickness of perovskite films based on the HPbI_3_–CH_3_NH_2_ reaction^20^ by varying the substrate temperature (Supplementary Figs. 4, 5, 6, Supplementary Table 2 and Supplementary Note 1). As a result, perovskite films with thickness about 1.1 μm are used as our benchmark for further studies. Partial substitution of iodine ions by chorine ions step depicted in Fig. 1a was achieved by reacting CH_3_NH_3_Cl (MACl) with HPbI_3_ to form HPbI_3_(Cl). Considering that MACl exists in the solid state at room temperature but sublimes at elevated temperatures (higher than 100 °C), the introduction of MACl can not only achieve a small amount of substitution of iodine ions by chorine ions, but also tune the morphology of HPbI_3_(Cl) films^27,35^. Top set in Fig. 1b shows the top-view scanning electron microscope (SEM) images of HPbI_3_(Cl) films prepared using HPbI_3_/MACl precursor solution with different MACl contents. It is found that upon increasing the MACl content, the size of voids between islands-like crystals in the HPbI_3_(Cl) films gradually decreases, and the crystals gradually turn from the hexagonal shape to the square shape and then to crystals with round edges (Supplementary Fig. 7). The enhanced coverage of HPbI_3_(Cl) films is expected to be beneficial for the subsequent CH_3_NH_2_ gas-based perovskite formation process.

**Table 1:** The SCLC results for FTO/perovskite/Au devices based on 1.1-μm-thick MAPbI_3_(Cl) and MAPbI_3_ films prepared by HPbI_3_(Cl)/CH_3_NH_2_ method, and MAPbI_3_(AS) film prepared by the antisolvent method

Samples	Thickness (μm)	*V*_FTL_ (V)	*N*_t_ (cm^−3^)	*μ* (cm^2^ V^−1^ s^−1^)
MAPbI_3_(Cl)	1.1	0.73	2.13×10^15^	1.12
MAPbI_3_	1.1	0.86	2.51×10^15^	0.83
MAPbI_3_ (AS)	0.45	0.85	1.48×10^17^	0.014

*V*_FTL_, trap-filled limit voltage; *N*_t_, defect density; *μ*, mobility

To further investigate the incorporation of chlorine into the HPbI_3_, we used X-ray diffraction (XRD) spectroscopy to characterize the obtained HPbI_3_(Cl) films. It is reported that the HPbI_3_ films show a hexagonal structure^20,34,36^. Upon increasing MACl content from 0 to 0.20, the low-angle peak around 11.7° shifts to a higher two theta value and the peak intensity increases significantly (Supplementary Fig. 8). This result suggests that the substitution of I^–^ ion with smaller Cl^–^ ion can shrink the crystal lattice, and HPbI_3_(Cl) still preserves the pseudo-hexagonal phase in the case of MACl content lower than 0.4. When the MACl content is increased to 0.40, the XRD peak intensity of the HPbI_3_(Cl) film at around 11.7° reduced substantially. This observation suggests that when the MACl content is too high, the crystal structure of HPbI_3_(Cl) cannot maintain the previous structure, which is also supported by the observed shape difference of crystal islands by SEM (Supplementary Fig. 7). This is because the large difference between the ionic radii of I^–^ and Cl^–^ makes the crystal structure unstable when the content of chlorine is too high. Such a phenomenon has also been observed in MAPbI_3−*x*_Cl_*x*_. When a small amount of Cl was incorporated, MAPbI_3−*x*_Cl_*x*_ can still maintain the pseudo-cubic phase^11,12^. However, a continuous solid phase of MAPbI_3−*x*_Cl_*x*_ theoretically cannot form at a high chlorine content due to the large size mismatch between chloride and iodide^30^. Besides, a slight splitting of the XRD peaks around 11.7° and the formation of MAPbI_3_ are observed for high MACl content of 0.70 and 1.00 cases. The former observation may be due to the incomplete removal of MACl or phase separation of HPbI_3_(Cl), and the latter observation suggests that the reaction of MACl with HPbI_3_ can form MAPbI_3_. The nonvisible perovskite peaks in HPbI_3_(Cl) films at low MACl content from 0.05 to 0.40 may be due to the low amount of MAPbI_3_ seeds and detection limit of XRD. Here, we can deduce that the possible reaction for the formation of HPbI_3_(Cl) at low MACl content up to 0.40 is as follows:

1 $$\begin{array}{l}{\mathrm{HPbI}}_{\mathrm{3}} + \left( {{{x}} + {{y}}} \right){\mathrm{MACl}} \to {\mathrm{H}}_{{{1 - x}}}{\mathrm{Pb}}_{{{1 - x}}}{\mathrm{I}}_{{{3 - 4x}}}{\mathrm{Cl}}_{{x}}\\ + {{x{\mathrm {MAPbI}}}}_{\mathrm{3}} + {{x{\mathrm {HI}}}}\,\left( {\mathrm{g}} \right) + {{y{\mathrm {MACl}}}}\,\left( {\mathrm{g}} \right)\end{array},$$

where the chlorine-incorporated HPbI_3_, i.e., H_1−*x*_Pb_1−*x*_I_3−4*x*_Cl_*x*_ is termed as HPbI_3_(Cl) hereafter; for MACl, *x* + *y* represents the initial amount of MACl in the HPbI_3_/MACl precusor solution, *x* represents the amount of MACl used for chlorine incorporation, *y* represents the amount of MACl that sublimes during thermal annealing.

**Fig. 2:**
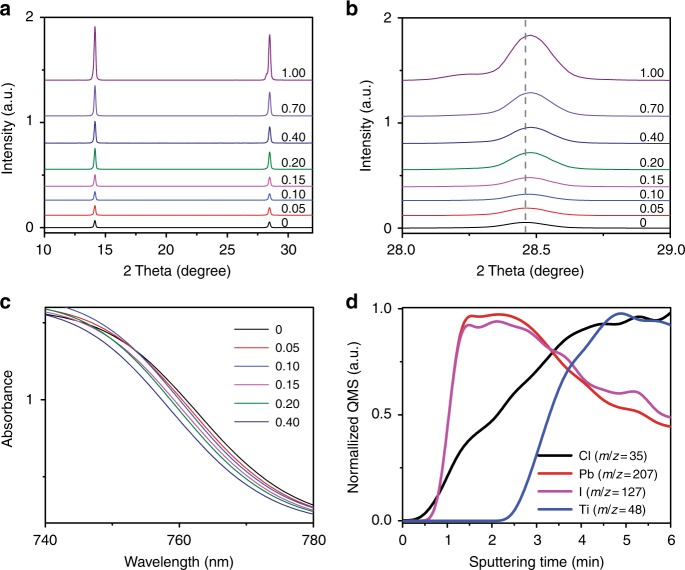
Partial substitution of iodine ions by chorine ions. **a**, **b** X-ray diffraction (XRD) patterns of MAPbI_3_ films prepared with different molar ratios of MACl versus HPbI_3_ in the HPbI_3_/MACl precursor solution. **c** UV−Vis spectra of MAPbI_3_ films prepared with different molar ratios of MACl versus HPbI_3_ in the HPbI_3_/MACl precursor solution. **d** SIMS profiles showing I (magenta), Pb (red), Cl (black), and Ti (blue) elements from the top to the bottom of the MAPbI_3_ film prepared with the molar ratio of MACl versus HPbI_3_ to be 0.10 in the HPbI_3_/MACl precursor solution

The as-prepared HPbI_3_(Cl) films were then exposed to CH_3_NH_2_ gas atmosphere to form perovskite via the reaction, CH_3_NH_2_ + H^+^ → CH_3_NH_3_^+^^20^. As shown in Fig. 1b bottom set, the apparent grain size of the MAPbI_3_(Cl) films gradually increases upon the increase of MACl content. However, too much MACl is found to cause significant deterioration of the film morphology, especially for the MACl content of 1.00 case (Supplementary Fig. 9). The structural properties of MAPbI_3_(Cl) films were further examined by XRD (Fig. 2a, b and Supplementary Fig. 10). All the samples show a preferred orientation along the (110) crystallographic plane, and the corresponding peak intensity becomes stronger with consecutive substitution of iodine ions by chorine ions. This preferable (110) crystal orientation is induced by the CH_3_NH_2_-based perovskite formation process^19,37^. The gradually increased peak intensity is ascribed to the effect of chlorine on perovskite film growth^27,35^. Furthermore, it was found that the low-angle XRD peak around 14.1° shifts to a higher two theta value and the peak intensity also increases upon increasing the MACl content. The same as the aforementioned discussion about the HPbI_3_(Cl), substitution of I^–^ by smaller-size Cl^–^ can also lead to the shrinkage of the crystal lattice of perovskite, which is consistent with the cell parameters variation (Supplementary Fig. 11). A slight splitting of the XRD peaks is also observed in the case of the MACl content of 1.00, in which the large size mismatch between chloride and iodide precludes the formation of a continuous solid phase of MAPbI_3−*x*_Cl_*x*_, and results in phase segregation and large cell parameter variation (Fig. 2b and Supplementary Fig. 11). The observed additional peak around 28.2 ° is likely associated with the hydrate phase of perovskite^38^.

Although MACl is introduced into precursor with nominal ratios of MACl/HPbI_3_ from 0.1 to 1, the remaining contents of chlorine in HPbI_3_(Cl) and MAPbI_3_(Cl) can be significantly lower than the nominal content of chlorine in precursor solution. This is due to the fact that MACl tends to sublime at elevated temperatures. Furthermore, chlorine tends to form volatile chlorine-containing species, which also tends to gradually diffuse and sublimate from the film, especially in the presence of water^25,27^. Thus, after thermal annealing in open air (relative humidity of about 40−60%) the remaining chlorine content in the resultant MAPbI_3_(Cl) films is much lower than the chlorine content in the precursor solution. The low content of chlorine in the MAPbI_3_(Cl) films is proven by the tiny change in absorption edge (Fig. 2c)^39^. It should be noted that the fluctuation of absorption for the case of MACl content of 0.70 and 1.00 may be due to the observed slight phase segregation and large roughness (Supplementary Fig. 12)^25,27,35,39^. Similar to chorine inclusion, phase segregation is also observed in the bromine inclusion case with high content, i.e., (MAPb(I_*x*_Br_1−*x*_)_3_) (0.1 < *x* < 0.8)^40^, in which perovskite undergo phase separation into iodide-rich and bromide-rich regions under light illumination^41^. In the case of bromine inclusion with low content, the existence of bromine is found to assist grain growth, improve stability, and device performance^40–42^.

To further confirm the existence of chorine in MAPbI_3_(Cl) films, we employed secondary-ion mass spectrometry (SIMS) to characterize the depth profile of chlorine in the perovskite film with MACl content of 0.1 (which gives the best device performance as discussed in the device performance section). As shown in Fig. 2d, the Cl depth profile exhibits a continuous and gradual increase, and then shows a significant spatial overlap on the Ti profile. This observation is consistent with other reports revealing that the distribution of chlorine within MAPbI_3_(Cl) is often inhomogeneous^25,43–45^. One possible cause responsible for the inhomogeneous distribution of chlorine is due to the tendency of chlorine to form volatile chlorine-containing species gradually diffusing and sublimating from the top surface of the film, especially in ambient air, resulting in a gradient increase of the Cl content from the film surface to bottom^25^. Although the XRD results of the MAPbI_3_(Cl) films do not show obvious peaks of PbCl_2_ or MAPbCl_3_, it is worth noting that in addition to incorporation into perovskite, the chlorine may form some chloride compounds that are nondetectable by XRD due to the detection limit or the amorphous nature of these compounds. Here, we can complete the second step of MAPbI_3_(Cl) formation reaction depicted in Fig. 1a as follows:

2 $$	{\mathrm{H}}_{{{1 - x}}}{\mathrm{Pb}}_{{{1 - x}}}{\mathrm{I}}_{{{3 - 4x}}}{\mathrm{Cl}}_{{x}} + {{x{\mathrm {MAPbI}}}}_{\mathrm{3}} \\ 	+ \left( {{{1 - x}}} \right)\,{\mathrm{CH}}_{\mathrm{3}}{\mathrm{NH}}_{\mathrm{2}} \to {\mathrm{MAPbI}}_{{{3 - x}}}{\mathrm{Cl}}_{{x}},$$

where the chlorine-incorporated perovskite, i.e., MAPbI_3−*x*_Cl_*x*_ is termed as MAPbI_3_(Cl) hereafter.

### Device performance

**Fig. 3:**
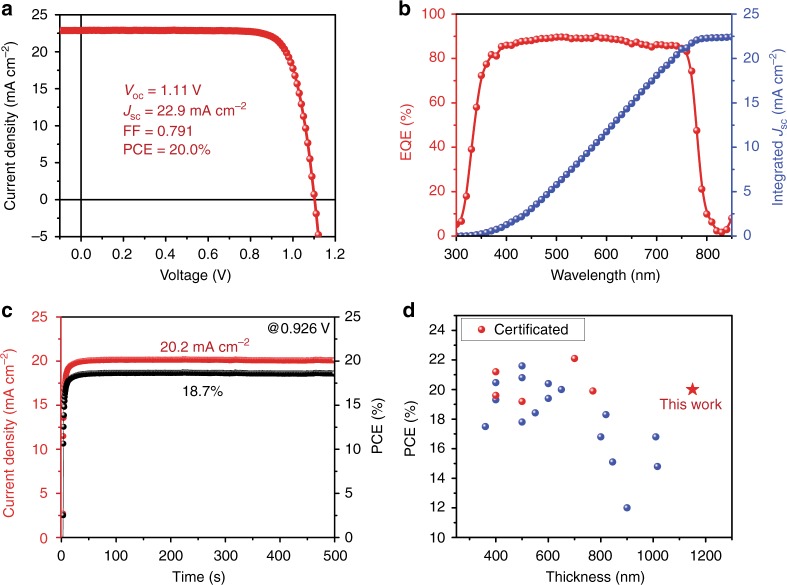
Photovoltaic performance. **a** Current density–voltage (*J*−*V*) curve for the best performing PSC with AM 1.5 radiation under ambient condition. **b** External quantum efficiency (EQE) spectrum and integrated *J*_SC_ for perovskite solar cells based on the 1.1-μm-thick MAPbI_3_(Cl) films. **c** Stabilized photocurrent density output by holding the voltage at the maximum power point (0.926 V) for PSCs based on 1.1-μm-thick MAPbI_3_(Cl) films. **d** Comparison of the PSCs and the corresponding perovskite film thickness in the representative reports and this work (Supplementary Table 1)

**Fig. 4:**
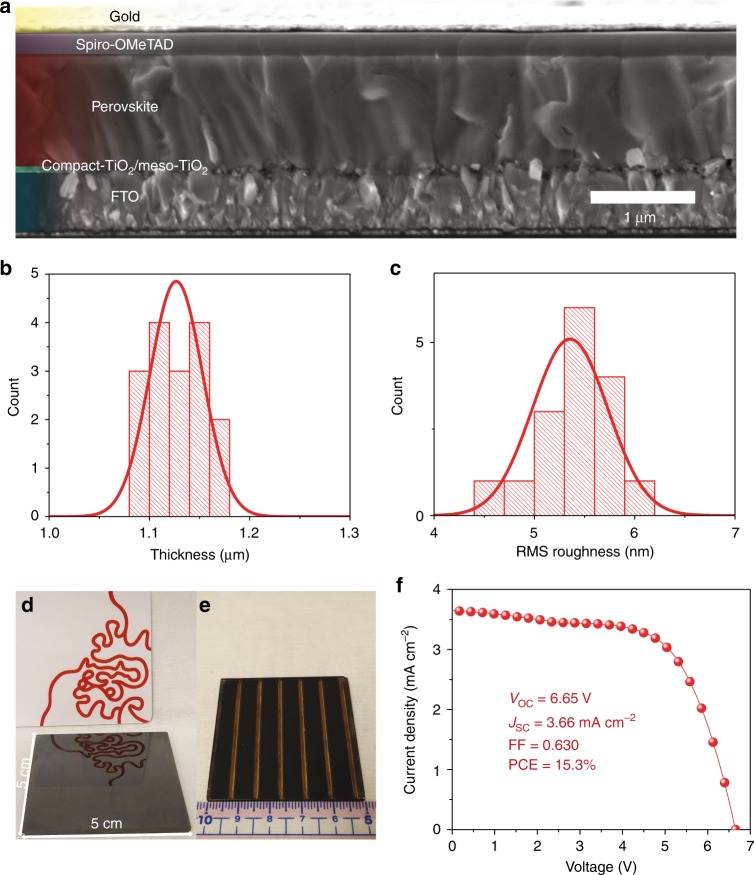
Solar cells and modules. **a** Cross-sectional-view SEM image of the PSC based on 1.1-μm-thick films. **b**, **c** Histograms of the thickness and root-mean-square (RMS) roughness of 16 1.1-μm-thick MAPbI_3_(Cl) samples, respectively. **d** Photograph of the 1.1-μm-thick MAPbI_3_(Cl) film on a 5 cm × 5 cm substrate with the mirror-like smooth surface. **e** Photograph of a 12.0 cm^2^ six-cell perovskite solar module fabricated using our new method. **f** *J*–*V* curve of the 5 cm × 5 cm perovskite module with an active area of 12.0 cm^2^ based on 1.1-μm-thick MAPbI_3_(Cl) films

To study the device performance, we implemented our smooth 1.1-μm-thick MAPbI_3_(Cl) films in the device configuration of fluorine-doped tin oxide (FTO) /compact TiO_2_/meso-TiO_2_/perovskite/spiro-OMeTAD/Au. Supplementary Fig. 13 and Supplementary Table 3 show the device performance results. The MACl content of 0.10 case delivers the best device performance (Supplementary Note 2) with an average current density (*J*_SC_) of 22.2 ± 0.3 mA cm^−2^, open-circuit voltage (*V*_OC_) of 1.09 ± 0.02 V, fill factor (FF) of 0.793 ± 0.015, and PCE of 19.1 ± 0.4%. As shown in Fig. 3a, a best PCE of 20.0% was achieved. The integrated *J*_SC_ from external quantum efficiency (EQE) is in excellent agreement with current density–voltage (*J*−*V*) measurements, with a discrepancy below 2% (Fig. 3b). The representative *J*–*V* curves under different scan directions indicate the negligible hysteresis effect (Supplementary Fig. 14). In addition, a stabilized PCE approaching 19% for device (with efficiency of 19.2% from *J*−*V* measurements) is also achieved by holding the voltage at the maximum power point for 500 s (Fig. 3c), which further confirms the negligible hysteresis effect. When compared with reports listed in Supplementary Table 1, the MAPbI_3_(Cl)-based devices in this work can also deliver a comparable high efficiency with perovskite film thickness over 1.1 μm as shown in Fig. 3d. This suggests that at least the 1.1-μm-thick MAPbI_3_(Cl) layer deposited here does not show a detrimental effect in carrier transport. To further inspect the devices, we performed the cross-sectional-view SEM (Fig. 4a). It is found that the grains extend across the entire absorber layer minimizing grain boundaries and providing facile pathways for efficient charge transport. All these results prove that we obtained high-quality thick perovskite films, and high efficiency PSCs can be fabricated based on these films.

Reproducibility is crucial for mass production of low-cost optoelectronic device applications. When compared with PSCs based on perovskite films fabricated using the antisolvent method, our 1.1-μm-thick MAPbI_3_(Cl) film-based devices show significantly improved reproducibility represented by a much smaller PCE standard deviation (decreasing from ±1.2% to ±0.4%, Supplementary Fig. 15 and Supplementary Table 4). The high reproducibility of device performance is attributed to the high reproducibility of 1.1-μm-thick MAPbI_3_(Cl) films. To confirm this point, we measured the thickness and roughness of 16 samples of the 1.1-μm-thick MAPbI_3_(Cl) films (Fig. 4b, c and Supplementary Figs. 16, 17). The average thickness and roughness together with their standard deviations are determined to be 1.13 ± 0.03 μm and 5.4 ± 0.4 nm, respectively. In addition, the device performance of 1.1-μm-thick MAPbI_3_(Cl) devices is higher than that of the devices based on 450-nm-thick perovskite film deposited by the antisolvent method. The higher efficiency for the former case is mainly due to the improved *J*_SC_ and FF, which is also supported by the EQE results in Supplementary Fig. 15. This improvement is not only due to the thicker film but also its improved film quality. Moreover, a slightly wider spectral response is observed for the 1.1-μm-thick MAPbI_3_(Cl) film-based devices. These results suggest that high-quality, thick perovskite films can benefit the enhancement of light harvesting capability, especially at the near-infrared region, towards better device performance.

Development of scalable fabrication processes is key to further industrialization of the PSC technology. We demonstrate that our new perovskite formation method is scalable and can be used to fabricate large-area solar modules. Figure 4d shows the optical photograph of 1.1-μm-thick MAPbI_3_(Cl) film on 5 cm × 5 cm substrates with the mirror-like smooth surface. It is also found that the film is much more uniform over the entire 5 cm × 5 cm substrate than that of antisolvent case (Supplementary Figs. 18, 19). We then fabricated six-cell modules (active area = 12.0 cm^2^, geometric fill factor = 48%) as shown in Fig. 4e. A best active area PCE of 15.3% under reverse scan was achieved with a *J*_SC_ of 3.66 mA cm^−2^, *V*_OC_ of 6.65 V, and FF of 0.630 (Fig. 4f), which is among the top PCE values obtained for perovskite solar modules with an active area greater than 10 cm^2^ ^35,46–56^. Furthermore, our new method shows good reproducibility in the large-area device fabrication with an average module PCE and standard deviation of 13.6 ± 0.8% (Supplementary Table 5), which is much better than that of the antisolvent case (8.6 ± 1.6%, Supplementary Fig. 20 and Supplementary Table 6). These results further confirm the excellent reproducibility of our 1.1-μm-thick MAPbI_3_(Cl) films on large scale, which is a key advantage for manufacturing in the realistic industrial large-scale setting. Note that small *J*–*V* hysteresis was observed for solar modules (Supplementary Fig. 21).

### Charge carrier transport behavior

**Fig. 5:**
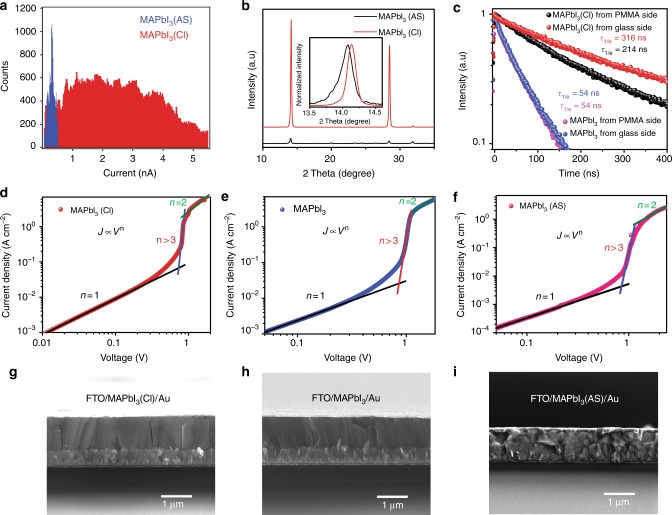
Charge carrier transport behavior. **a** The light-induced current extracted from the c-AFM results and **b** XRD spectra for 1.1-μm-thick MAPbI_3_(Cl) film prepared by the HPbI_3_(Cl)/CH_3_NH_2_ method and MAPbI_3_(AS) film prepared by the antisolvent method. **c** Time-resolved photoluminescence (TRPL) decay for 1.1-μm-thick MAPbI_3_(Cl) and MAPbI_3_ films prepared by the HPbI_3_(Cl)/CH_3_NH_2_ method. **d**–**f** Current density–voltage (*J*−*V*) characteristics of devices with FTO/perovskite/Au configuration and **g**–**i** corresponding cross-sectional-view SEM images utilized for estimating the trap density and mobility for 1.1-μm-thick MAPbI_3_(Cl) and MAPbI_3_ films prepared by the HPbI_3_(Cl)/CH_3_NH_2_ method, and MAPbI_3_(AS) film prepared by the antisolvent method. Scale bars: 1 μm

As shown in Fig. 3d, we obtained high efficiencies with 1.1-μm-thick perovskite films. The promising device performance inspired us to conduct systematical investigations of charge transport properties of our PSC devices to find out what factors lead to the high device performance. We first performed conductive atomic force microscopy (c-AFM) measurements on the 1.1-μm-thick MAPbI_3_(Cl) film and perovskite film prepared by the antisolvent method (denoted as MAPbI_3_(AS)). It was found that the former shows lower roughness (Supplementary Note 3) and rounded large grains with almost four times larger grain size, and its corresponding fewer grain boundaries are expected to reduce the defects density (Supplementary Fig. 22). Moreover, the 1.1-μm-thick MAPbI_3_(Cl) film also shows nearly an order of magnitude higher light-induced current (Fig. 5a), which indicates its good electrical conductivity. This observation can be ascribed to the improved crystallinity of the MAPbI_3_(Cl) films with preferential (110) orientation (Fig. 5b), as a result of synergistic effect of CH_3_NH_2_ gas^19,57^ and partial substitution of I^−^ by Cl^−^^11,12^.

In addition to the improvement in terms of the film morphology and conductivity, the defect nature in perovskite films may also play a role in influencing charge carrier transport properties^58^. To investigate radiative and nonradiative charge carrier recombination channels within perovskite films, we carried out time-resolved photoluminescence (TRPL) measurements on glass/perovskite/polymethyl methacrylate (PMMA) samples (Supplementary Fig. 23). Figure 5c shows the TRPL decay curves obtained from both the glass side and PMMA side for the 1.1-μm-thick perovskite films with (MAPbI_3_(Cl)) and without (denoted as MAPbI_3_) chlorine incorporation. Considering the limited penetration depth (tens of nanometers) of the light source, the PL emission signal only contains the charge carrier information on the surface layer that is facing the light source^59^. The time constants (*τ*) are calculated to be 54 ns for the MAPbI_3_ film no matter whether the laser light incidents from the PMMA side or the glass side, which indicates that perovskite on the bottom and top of film has nearly the same charge carrier properties. In the MAPbI_3_(Cl) case, the PL lifetime obtained from the glass side is longer than that obtained from the PMMA side. This observation suggests that perovskite on the bottom has better charge carrier properties than the perovskite on the top in MAPbI_3_(Cl) film. This observation may be correlated with the inhomogeneous distribution of chlorine within MAPbI_3_(Cl) film, where chlorine within a certain range of concentrations can benefit charge carrier properties. Besides, the MAPbI_3_(Cl) film shows longer PL lifetimes than that of the MAPbI_3_ film. This improvement can be ascribed to the better crystallinity and a lower defect density of the MAPbI_3_(Cl) film^60^.

To quantitatively evaluate the defect density, we fabricated sandwich devices by inserting the perovskite films between FTO and gold, and characterized the evolution of the space-charge-limited current (SCLC) for different biases, as shown in Fig. 5d−i. In general, the presence of mobile species in perovskite can lead to complication in interpreting the SCLC measurement results, and often precludes a robust quantitative analysis based on such measurements. On the other hand, we observed low hysteresis on the basis of *J*–*V* measurements, which indicates a negligible effect of mobile species. Thus, in this case we can safely deduce defects density (*N*_t_) within perovskite films based on the SCLC measurements according to the equation^13,61^:

$$N_{\mathrm{t}} = \frac{{2\varepsilon \varepsilon _0V_{{\mathrm{TFL}}}}}{{eL^2}}$$

where *ε* and *ε*_0_ are the dielectric constants of perovskite and the vacuum permittivity, respectively, *V*_TFL_ is trap-filled limit voltage_,_ *L* is the thickness of the perovskite films, and *e* is the elementary charge. We estimated the defect density to be 2.13×10^15^ and 2.51×10^15^ cm^−3^ for the 1.1-μm-thick MAPbI_3_(Cl) film and MAPbI_3_ film, respectively, prepared by the HPbI_3_(Cl)/CH_3_NH_2_ method (Table 1). These defect density values are significantly lower than antisolvent case (1.48×10^17^ cm^−3^). The similar magnitude in terms of defects densities derived from the SCLC measurements in contrast with the clear difference in terms of lifetime derived from PL measurements is possibly due to the fact that the former measurement gives the defect information in the entire films, and the latter measurements only provides the defect information for perovskite on the top or bottom of the film. When the bias voltage further increases in the SCLC curves, the current shows a linear relationship with the square of the voltage, where we can deduce the mobility (*μ*) from the Mott−Gurney law^61^:

$$J_{\mathrm{D}} = \frac{{9\varepsilon \varepsilon _0\mu V^2}}{{8L^3}},$$

The mobility are estimated to be 1.12 and 0.83 cm^2^ V^−1^ s^−1^ for the 1.1-μm-thick MAPbI_3_(Cl) film and MAPbI_3_ film, respectively. The much higher carrier conductivity is consistent with the higher device performance of the MAPbI_3_(Cl) case, which again verifies the positive effect of small content chlorine incorporation on the optoelectronic properties of perovskite films. In addition, these mobility values around 1 cm^2^ V^−1^ s^−1^ are two orders of magnitude higher than that in antisolvent case (0.014 cm^2^ V^−1^ s^−1^), which further confirms the excellent charge carrier transport properties of CH_3_NH_2_ gas-based perovskite films. The improved carrier properties of the 1.1-μm-thick MAPbI_3_(Cl) films when implemented into PSCs is also verified by impedance spectroscopy characterization of devices under light illumination (Table 1 Supplementary Fig. 24 and Supplementary Note 4).

### Device stability and perovskite film stability

**Fig. 6:**
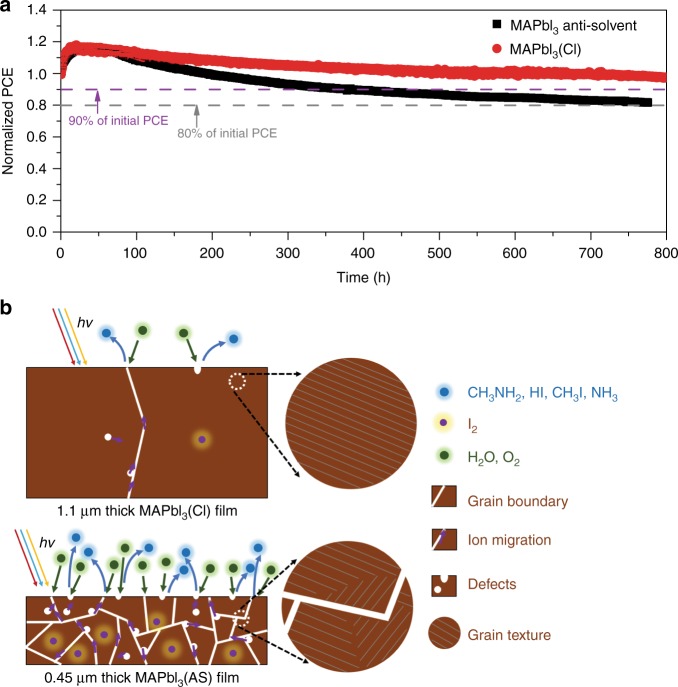
Device stability and film stability. **a** Operational stability of un-encapsulated PSCs based on 1.1 μm thick MAPbI_3_(Cl) films and perovskite films deposited via the antisolvent method examined under continuous full-sun illumination in dry nitrogen atmosphere. **b** Factors influence film stability of perovskites

Operational stability is another key aspect of PSCs. We studied the stability of the small size devices under the operation conditions (Fig. 6a). It was found that the 1.1-μm-thick MAPbI_3_(Cl)-based devices showed much better stability than the MAPbI_3_(AS) devices. Especially, the MAPbI_3_(Cl) devices maintained over 90% of their initial performance for 800 h under continuous light illumination with maximum power point tracking (MPPT). While the PCEs of the MAPbI_3_(AS) devices decreased to 90% of their initial performance after approximately 300 h. An initial increase of PCEs for both samples is observed in the first 20 h of the stability test. A similar phenomenon was also observed previously^55,62^, and was ascribed to the effects such as elevated temperature (approximately 46 °C), light or field induced ion movement, light-induced traps formation, interfacial charge accumulation^55^, or spiro-OMeTAD conductivity variation^63^. In addition, both devices showed a fast, exponential decay region after reaching the highest performance points, which is followed by a slower linear decay^64^. By fitting the linear region, the MAPbI_3_(Cl) devices exhibit a linear slope of −0.0014% h^−1^, which is 30% slower than the antisolvent case with a linear slope of −0.0020% h^−1^. Using these slope values, it is expected to take 1660 and 870 h of operation to reach the T_80_ points for MAPbI_3_(Cl) devices and the MAPbI_3_(AS) devices, respectively. The nearly doubled T_80_ parameters for the MAPbI_3_(Cl) devices suggests the excellent long-term stability of the cells based on our Cl-incorporated thick films. Note that the stability measurements in this study were conducted under operation conditions, i.e., continuous illumination, MPPT, dry N_2_ gas environment simulating the case of encapsulation, and therefore the results obtained here can be used as a realistic indicator of the device operation lifetime of our PSCs. It should be noted that all of the components in PSCs play an important role in device stability. Considering that MAPbI_3_(Cl) devices and MAPbI_3_(AS) devices are assembled using the same device configuration, the impact of charge selective layers and electrode on device stability can be excluded out. Hence, the significant stability difference between MAPbI_3_(Cl) devices and MAPbI_3_(AS) devices is most likely caused by the quality of the perovskite films such as crystallinity, grain size and/or defects, etc. ^13,22,34^.

To reveal the underlying mechanism responsible for the improved stability, we prepared four kinds of films, i.e., film A: 1.1 μm MAPbI_3_(Cl) film prepared by the HPbI_3_(Cl)/CH_3_NH_2_ method; film B: 1.1 μm MAPbI_3_ film by the HPbI_3_/CH_3_NH_2_ method; film C: 0.5 μm MAPbI_3_(Cl) films prepared by the HPbI_3_(Cl)/CH_3_NH_2_ method; film D: 0.45 μm MAPbI_3_(AS) films prepared by the antisolvent method. We tested the stability of these films under light illumination, a high relative humidity of about 100% and gentle thermal environment of 50 °C (denoted as LHT) as shown in Supplementary Fig. 25. It is found that that the stability of these four films is in the order film A > film B > film C > film D (Supplementary Figs. 25 to 28). The improved stability of film A is firstly attributed to its improved grain crystallinity and larger grain size, which can be clearly seen by comparing the XRD peak intensity and full width half maximum of fresh film A with fresh film D (Supplementary Fig. 28b, c and Supplementary Note 5). This is because the ions in highly crystalline perovskite films have higher chemical binding energies to prevent themselves from migrating, which benefits light and thermal stability^65^. This is confirmed by SEM results shown in Supplementary Fig. 29. We found that the decomposition of perovskite mainly started from grain boundaries for all films after 1 h of LTH testing, where H_2_O and O_2_ can easily react with perovskite films due to the facts that (1) chemical binding at grain boundaries with low crystallinity is much weaker than that within crystals and (2) the severe ion migration in low crystallinity region especially at grain boundaries facilitates degradation (Fig. 6b)^65^. When further comparing film A with film B, film A shows better crystallinity, larger grain size, and also better stability (Supplementary Figs. 26 to 30). In addition, better stability of films A and B than thinner films C and D also verifies that thicker films are more advantageous in achieving better stability because the extra top layer of the compact high-quality film in the thicker films naturally helps as a protection layer to protect the film underlying the top layer. Besides, previous reports have demonstrated that often a tiny amount of I_2_ existing in perovskite films can cause severe degradation of perovskite^13,66^. While, CH_3_NH_2_ used here provides an alkaline condition to suppress the formation of I_2_ during the perovskite formation process and/or reduce the I_2_ amount during the initial degradation of iodine containing perovskite films (Fig. 6b and Supplementary Fig. 28f), which may contribute to the better stability of films A, B and C than film D^13,66^.

## Discussion

A simple perovskite formation method based on fast reaction of chlorine-incorporated HPbI_3_ and CH_3_NH_2_ has been developed to fabricate high-quality, over 1 μm thick and stable CH_3_NH_3_PbI_3_(Cl) perovskite films in ambient condition. The resultant films can not only enable high efficiencies of 19.1 ± 0.4% (champion PCE = 20.0%) for small size PSCs and 13.6 ± 0.8% (champion active area PCE = 15.3%) for 5 cm × 5 cm perovskite solar modules, but also deliver excellent device reproducibility. In addition, the resultant un-encapsulated small size PSCs exhibit an excellent T_80_ lifetime exceeding 1600 h under continuous light illumination with MPPT in dry N_2_ environment. The excellent device stability is mainly a result of the excellent films stability. Our study not only provides a highly reproducible method to fabricate PSCs and modules with enhanced efficiency and stability, but also offers the in-depth understanding for the underlying mechanisms responsible for device stability improvement. The encouraging device performance and excellent stability in this work points out to a promising direction, i.e., use of thick absorber films to realize PSCs and modules with high efficiency, reproducibility, and stability.

## Methods

### Materials

All reagents were used as received without further purification, including PbI_2_ (99.99%, TCI), hydroiodic acid (57 wt% in H_2_O, distilled, stabilized, 99.95%, Sigma-Aldrich), chlorobenzene (99.8%, Wako), methylamine (40% in methanol, TCI), 2,2′,7,7′-tetrakis(*N*,*N*-di-p-methoxyphenylamine)-9,9′-spirobifluorene (spiro-OMeTAD, Merck), *N*,*N*-dimethylformamide (DMF, 99.99%, Sigma-Aldrich), methylammonium chloride (Wako), ethanol (Wako), HI (57%, Alfa Aesar), 4-*tert*-butylpyridine (99.9%, Sigma-Aldrich), acetonitrile (99.9%, Sigma-Aldrich), titanium diisopropoxide bis(acetylacetonate) (75 wt% in isopropanol, Sigma-Aldrich), *n*-butyl alcohol (Sigma-Aldrich), FTO glasses (7−8 Ω sq^−1^, Opvtech.), TiO_2_ (DyeSol, 30 NR-D).

Synthesis of HPbI_3_: The HPbI_3_ crystals were prepared using an antisolvent vapor-assisted crystallization method as shown in Supplementary Fig. 2. Briefly, 11 g PbI_2_ and 7 g of HI were mixed and dissolved in 17 g of DMF to form an HPbI_3_ solution. The HPbI_3_ solution was heated at 100 °C in chlorobenzene vapor environment for 24 h. During this heat-treatment, chlorobenzene diffuses into the HPbI_3_/DMF solution and reduces the solubility of HPbI_3_. Light yellow needle-like HPbI_3_ crystals were formed, which were collected and washed with chlorobenzene/DMF (volume ratio = 3:1) for three times and then washed with ethanol for three times. Finally, the HPbI_3_ crystals were dried at 60 °C for 24 h under vacuum.

### Fabrication of perovskite films and solar cells

To study the dependence of the film thickness on preparation conditions, the HPbI_3_ films are obtained by spin-coating HPbI_3_ precursor (60 wt% HPbI_3_ in DMF) at 5000 rpm for 30 s on substrates under different temperatures (room temperature, 60, 70, 80, 90, 100 °C) in ambient air condition. For chlorine-incorporated perovskite deposition, the precursor solutions containing different molar ratios of MACl/HPbI_3_ (0, 0.05, 0.10, 0.15, 0.20, 0.40, 0.70, 1.00) are deposited on substrates preheated at 90 °C at 5000 rpm for 30 s. After thermal annealing at 100 °C for 5 min to remove the solvent, part of the MACl and also part or all of the formed HI during this process, the obtained films react with CH_3_NH_2_ gas to form perovskite^20,22^. Then the obtained perovskite films are annealed at 100 °C for 5 min in ambient air condition before device fabrication or other characterization. A 30 μL of spiro-OMeTAD was spin coated on the perovskite film at 3000 rpm for 30 s, where a spiro-OMeTAD/chlorobenzene (72.3 mg mL^−1^) solution was employed with the addition of 17.5 μL Li-TFSI/acetonitrile (520 mg mL^−1^), and 28.8 μL 4-*tert*-butylpyridine. Finally, a gold layer with a thickness of 100 nm was deposited as the counter electrode on the top of spiro-OMeTAD layer through shadow masks via thermal evaporation under high vacuum (5×10^−5^ Torr).

### Characterization

Current–voltage (*J*–*V*) characteristics of PSCs are measured under one sun illumination (AM 1.5 G, 100 mW cm^−2^, calibrated using a Newport reference Si-cell, Oriel Instruments Model Number 90026564, 2 × 2 cm^2^) using a solar simulator (Newport Oriel Sol 1A, Xenon-lamp, USHIO, UXL-150SO) and a Keithley 2400 source meter in ambient air at about 25 ^o^C and a relative humidity of about 40−60%. The small size PSCs were measured using a 0.1 cm^2^ metal mask. No mask is used for measurement of perovskite modules. The active area of perovskite modules is 12.0 cm^2^, determined by the overlap areas of top and bottom electrodes described in our previous publication^46^. All the *J*−*V* curves are measured under reverse scan with a scan rate of 0.25 V s^−1^ without preconditioning unless otherwise specified. For stability measurements, the cells were subsequently loaded in our home-designed environmental chamber coupled with a solar simulator (Peccell PEC-L01, AM1.5G) and source meter (Keithley 2401) controlled by a LabView program allowing automatic sequential measurements on the devices with adjustable acquisition time intervals. To simulate continuous solar cell operation an active bias voltage was applied to the cells maintaining the solar cell operation at the maximum power point. The devices were kept at the maximum power output voltage during the intervals between consecutive measurements. No UV-filters were used, i.e., the UV component is included in illumination. The stability measurement was performed under nitrogen box with a relative humidity below 5%. EQE measurements were performed on an Oriel IQE 200 in DC mode.

SEM measurements was carried out in scanning electron microscope (Helios NanoLab G3 UC, FEI). XRD measurements were carried out in a Bruker D8 Discover instrument (Bruker AXS GmbH, Karlsruhe, Germany) equipped with Cu wavelength *λ* = 1.54 Å X-ray source operated at 1600 W and Goebel mirror. Data were collected from 5 to 60 two theta degrees with a 0.02 degrees step. Experimental data were fitted to obtain unit cell phase parameters using the Profex/BGMN software (v.3.12.0, http://profex.doebelin.org/). TRPL was acquired using the time-correlated, single-photon counting technique (Hamamatsu, C10627), and excitation was provided by a femtosecond mode-locked Ti:sapphire laser (Spectra-Physics, MAITAI XF-IMW) at 450 nm with an average power at 8 MHz of 0.74 mW. In TRPL, perovskite films were covered with PMMA on the top of the perovskite layer to exclude/minimize the influence of ambient air (especially the influence from H_2_O and O_2_)^67–70^. SIMS (Kratos Axis ULTRA) equipped with quadrupole mass spectrometer (HAL 7, Hiden Analytical) was used to collect the elemental signal in positive ion detection mode (PID). For sputtering in SIMS, 3 keV Ar^+^ primary beam with a 10 mA current and diameter of 100 µm were utilized. The beam was set at an angle of 45° with respect to the sample surface normal. The spectrometer was operated at a pressure of 10^−8^ Torr. The c-AFM measurement of the films was performed in contact-mode (MFP-3D series, Asylum Research) in ambient conditions (35% RH, 22 °C). Top side illumination was generated with a halogen lamp. Chromium/platinum-coated cantilevers with a nominal spring constant of 0.2 N m^−1^ were used to collect the photo-generated currents. The SCLC data were collected with a semiconductor characterization system in N_2_ (4200-SCS, Keithley). Absorbance was measured using a UV−Vis spectrometer (JASCO Inc., V-670). Impedance Spectroscopy measurements had been recorded with an Autolab PGSTAT204 potentiostat equipped with a frequency response analyzer module FRA32, AC perturbation was set to 10 mV and frequency ranged between 1 MHz to 0.5 Hz. The measurements were done in N_2_ environment with less than 0.1% relative humidity, and Oriel VeraSol-2class AAALED light source simulating AM 1.5 spectra.

## Electronic supplementary material

Supplementary Information

## Data Availability

The data that support the findings of this study are available from the corresponding author on reasonable request.
