# Supplementary material for: Gas-solid reaction based over one-micrometer thick stable perovskite films for efficient solar cells and modules
Source: Nat Commun. 2018 Sep 24;9:3880. doi: 10.1038/s41467-018-06317-8 (PMC6155243; doi:10.1038/s41467-018-06317-8)
Supplement: Supplementary file 1 — Supplementary Information [file 41467_2018_6317_MOESM1_ESM.pdf]

Supplementary Information

Gas-Solid Reaction Based Over One-Micrometer Thick Stable Perovskite Films for Efficient Solar Cells and Modules

Zonghao Liu[†], *Longbin Qiu*[†], *Emilio J. Juarez-Perez*[†], *Zafer Hawash*[†], *Taehoon Kim*[†], *Yan Jiang*[†], *Zhifang Wu*[†], *Sonia R. Raga*[†], *Luis K. Ono*[†], *Shengzhong (Frank) Liu*[‡,§], *Yabing Qi**[,†]

[†] Energy Materials and Surface Sciences Unit (EMSSU), Okinawa Institute of Science and Technology Graduate University (OIST), 1919-1 Tancha, Onna-son, Kunigami-gun, Okinawa 904-0495, Japan

[‡] Key Laboratory of Applied Surface and Colloid Chemistry, Ministry of Education, Shaanxi Key Laboratory for Advanced Energy Devices, Shaanxi Engineering Lab for Advanced Energy Technology, School of Materials Science and Engineering, Shaanxi Normal University, Xi'an 710119, China

[§] Dalian National Laboratory for Clean Energy, iChEM, Dalian Institute of Chemical Physics, Chinese Academy of Sciences, 457 Zhongshan Road, Dalian, 116023, China

Corresponding author: *Yabing.Qi@OIST.jp (Y.B.Q.)

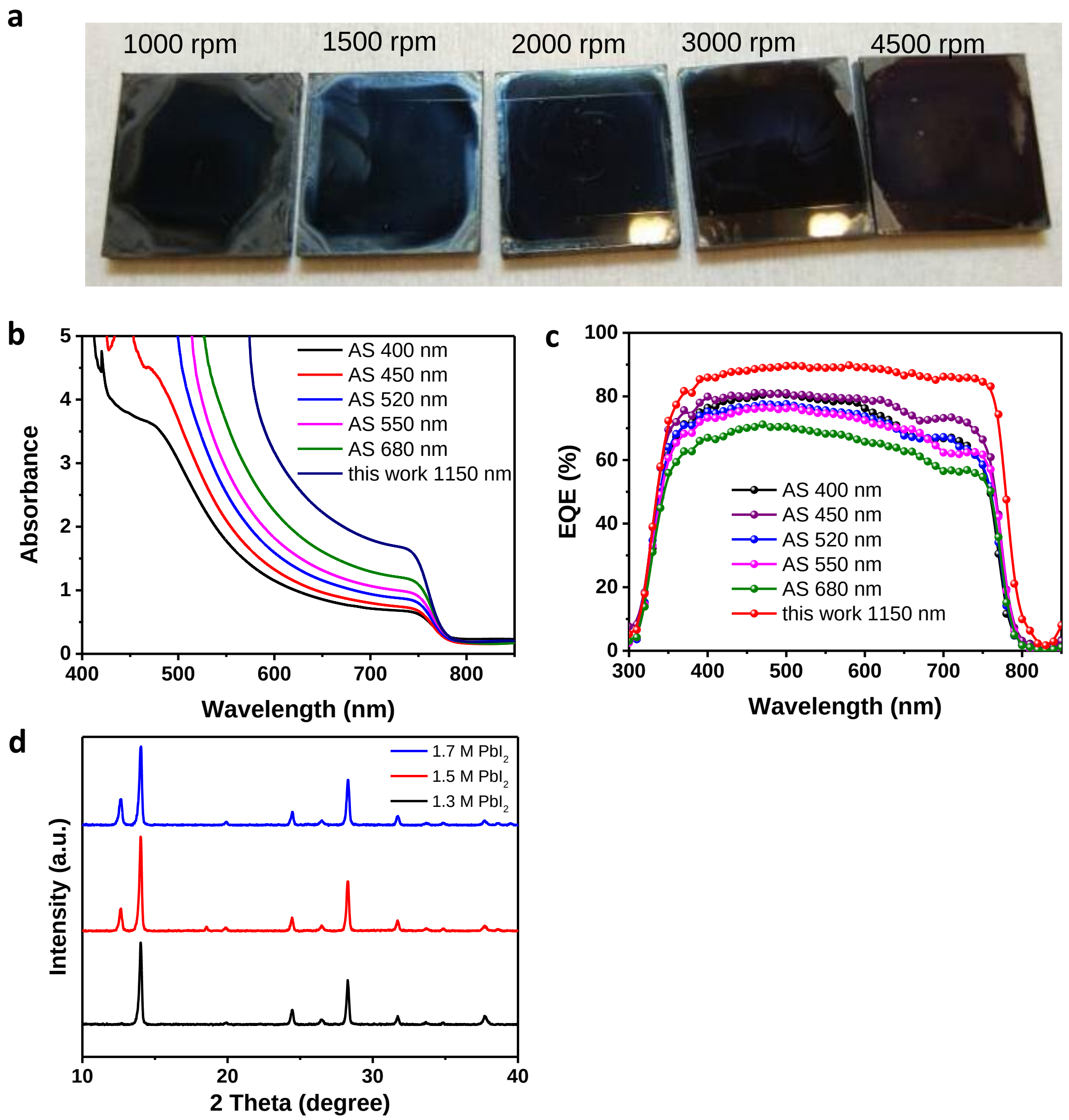

Supplementary Figure 1 | (a) Optical images, (b) UV-vis spectra and (c) EQE response of $MAPbI_3$(AS) films prepared with different spin coating speeds from 1.4 M $MAPbI_3$ solution via the anti-solvent (AS) method.[1] It was found that it is difficult to control the morphology when preparing thick perovskite films with the conventional AS method. The EQE results showed that when increases the thickness of perovskite film deposited by AS method from 400 nm to 450 nm, the light response of solar cell is enhanced due to improved light absorption, especially at the spectrum region from 600 to 800 nm. However, further increases the thickness of perovskite film does not increase the EQE response, this is due to the inferior film quality induced charge recombination and poor charge collection.(d) X-ray diffraction (XRD) patterns of FA/MA perovskite films based on different PbI_2 concentrations via the two-step inter-diffusion method.[2] It was found that it is difficult to achieve complete transformation of PbI_2 to perovskite when preparing thick perovskite films with the two-step method. These results suggest that it is difficult to prepare thick perovskite films with commonly used preparation methods to achieve high efficiency perovskite solar cells.

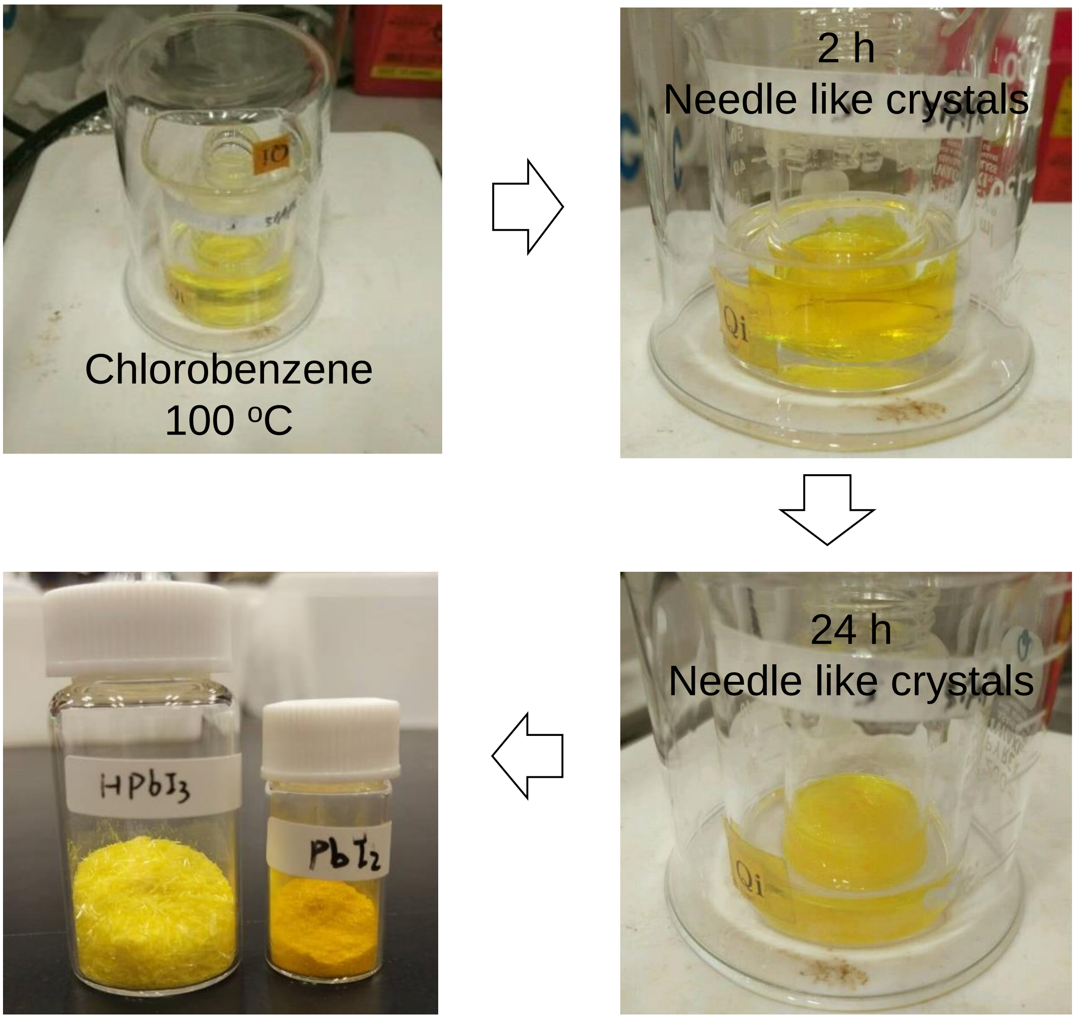

Supplementary Figure 2 | Optical images showing the synthesis of $HPbI_3$ crystals.

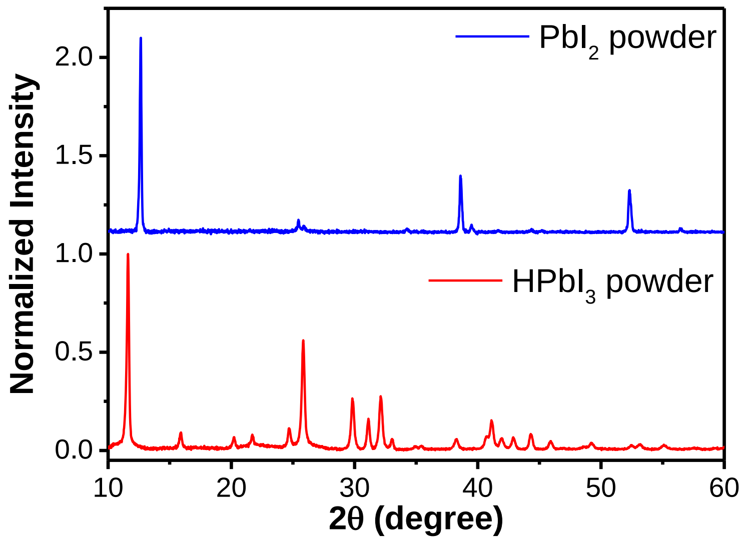

Supplementary Figure 3 │ X-ray diffraction (XRD) patterns of $HPbI_3$ crystals and PbI_2 powder.

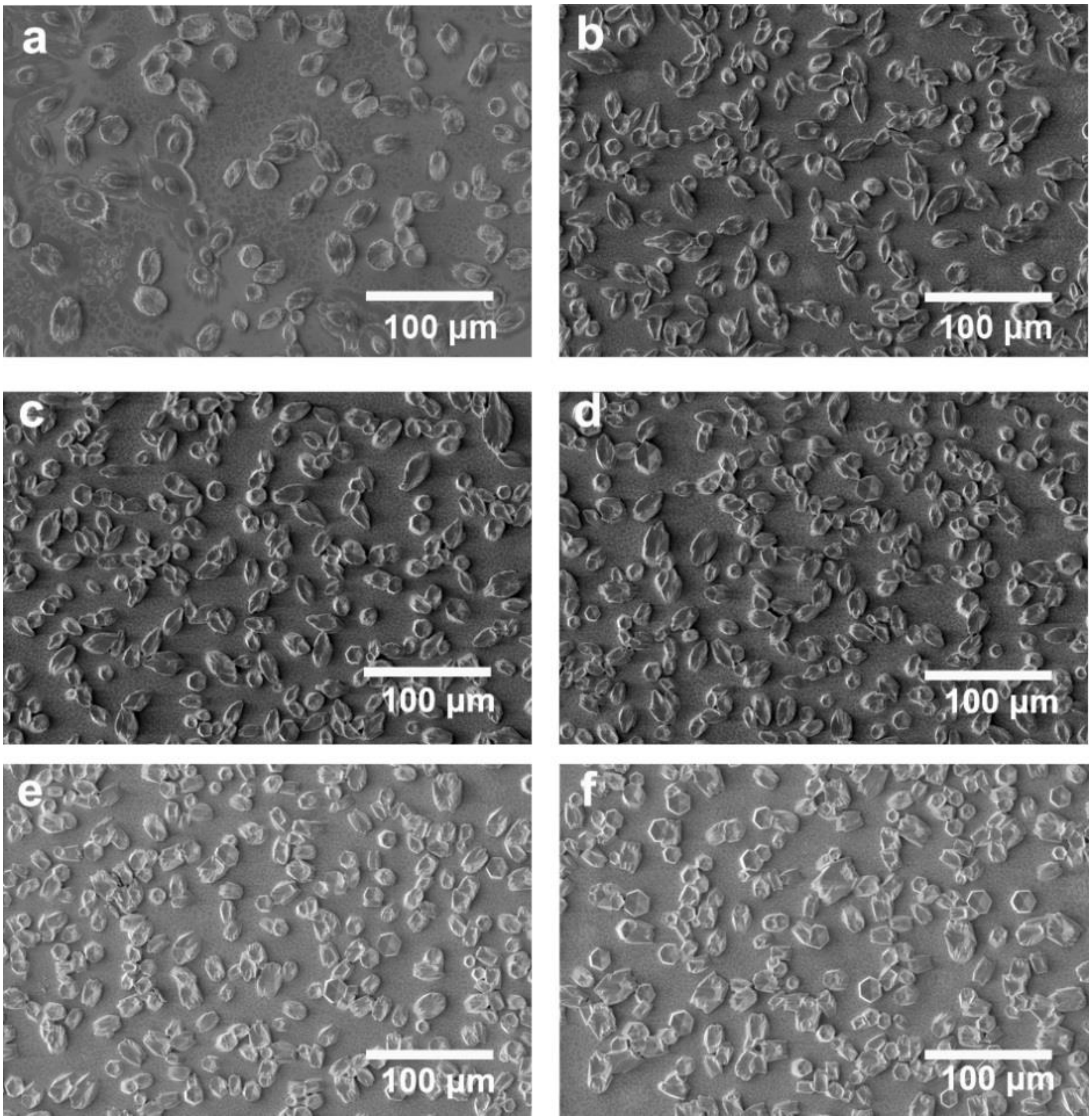

Supplementary Figure 4 │ Top-view SEM images of the $HPbI_3$ films prepared by the spin-coating $HPbI_3$ precursor solution under different substrate temperatures: a) room temperature, b) 60 °C, c) 70 °C, d) 80 °C, e) 90 °C, f) 100 °C, respectively.

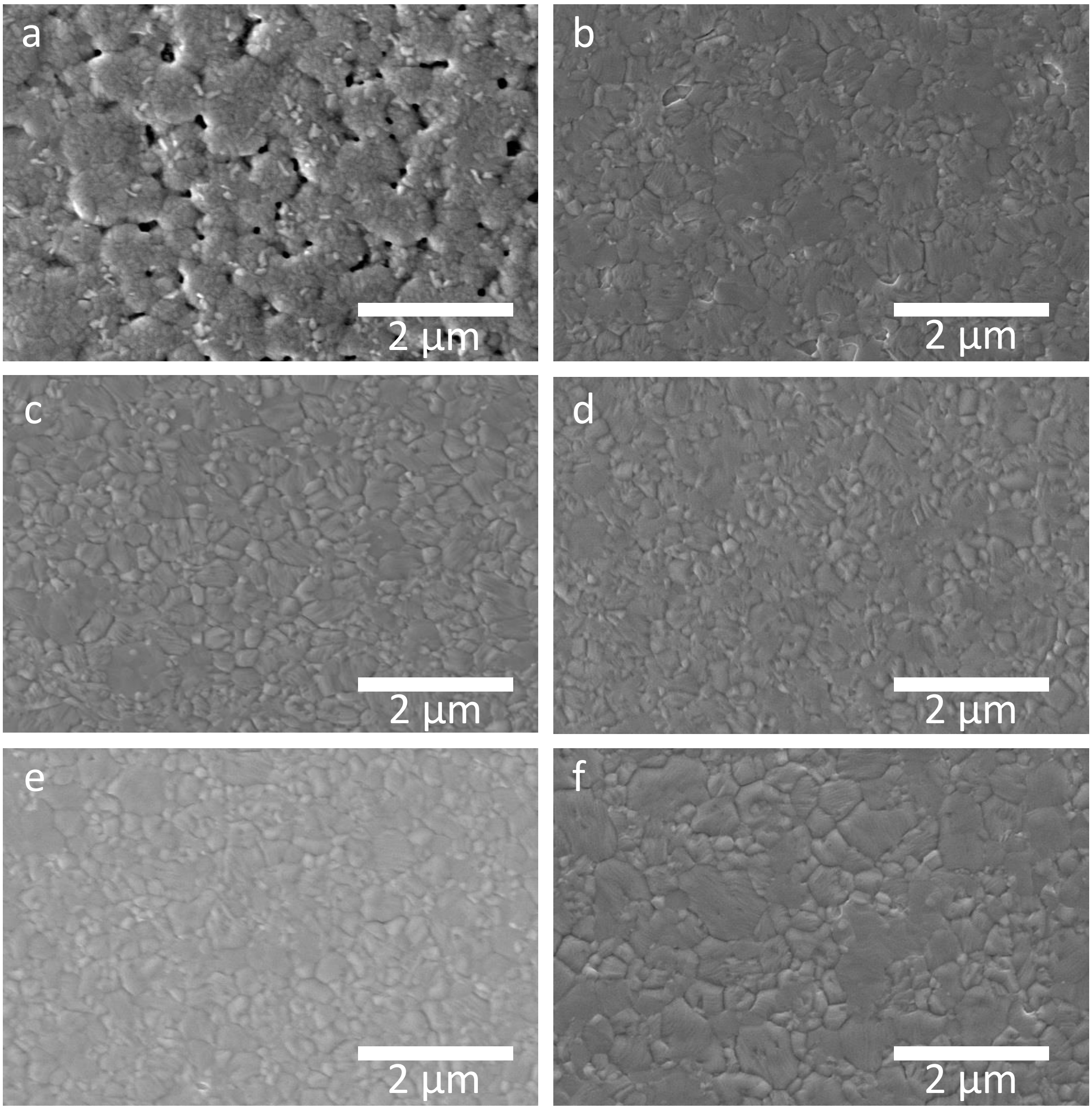

Supplementary Figure 5 │ Top-view SEM images of the $MAPbI_3$ films converted from the $HPbI_3$ films prepared by spin-coating the $HPbI_3$ precursor solution under different substrate temperatures: a) room temperature, b) 60 °C, c) 70 °C, d) 80 °C, e) 90 °C, f) 100 °C, respectively.

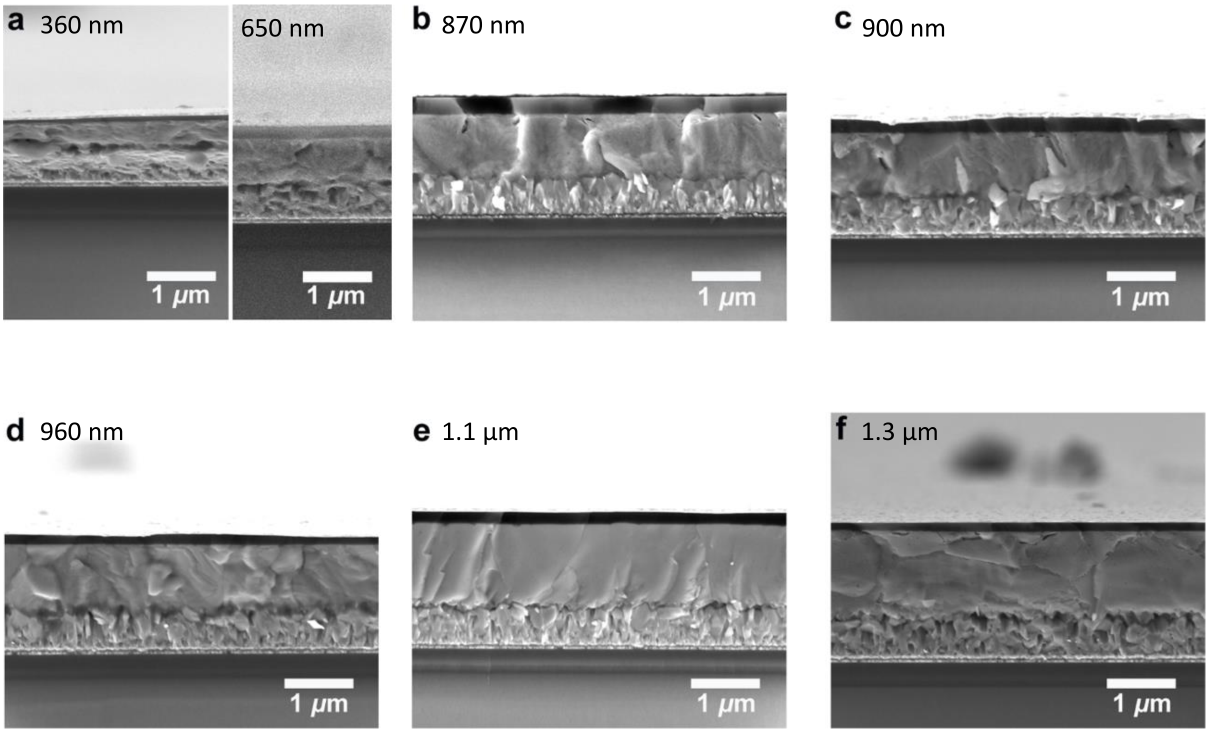

Supplementary Figure 6 | Cross-sectional-view SEM images of the perovskite films with different film thicknesses as indicated. The $MAPbI_3$ films were converted from the $HPbI_3$ films prepared by spin-coating the $HPbI_3$ precursor solution under different substrate temperatures: a) room temperature, b) 60 °C, c) 70 °C, d) 80 °C, e) 90 °C, f) 100 °C, respectively.

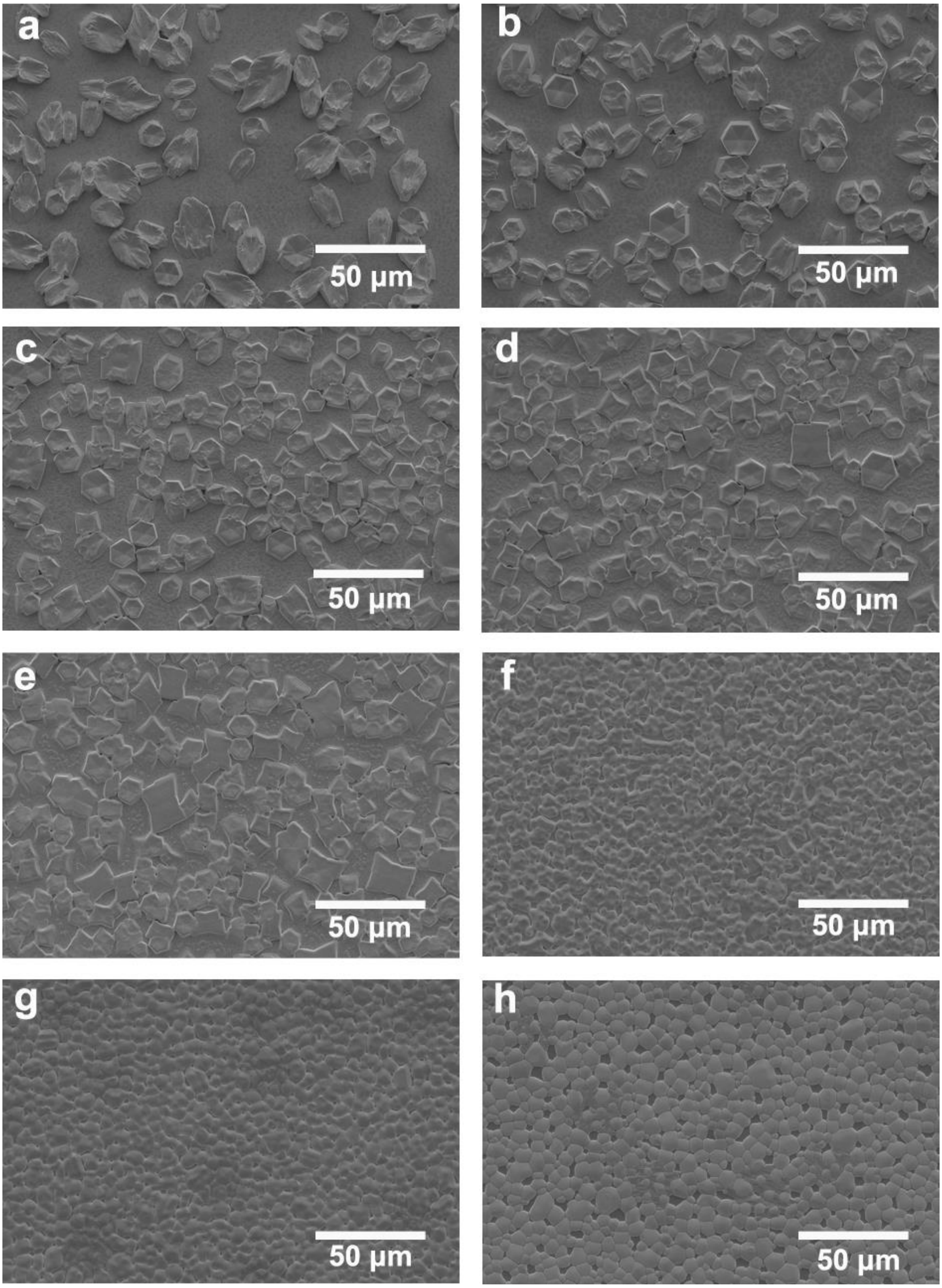

Supplementary Figure 7 | Top-view SEM images of the $HPbI_3(Cl)$ films prepared with the molar ratios of MACl versus $HPbI_3$ of (a) 0, (b) 0.05, (c) 0.10, (d) 0.15, (e) 0.20, (f) 0.40, (g) 0.70, (h) 1.00, respectively. These ratios refer to the ones used in the $HPbI_3$/MACl precursor solution. Scale bar: 50 μm.

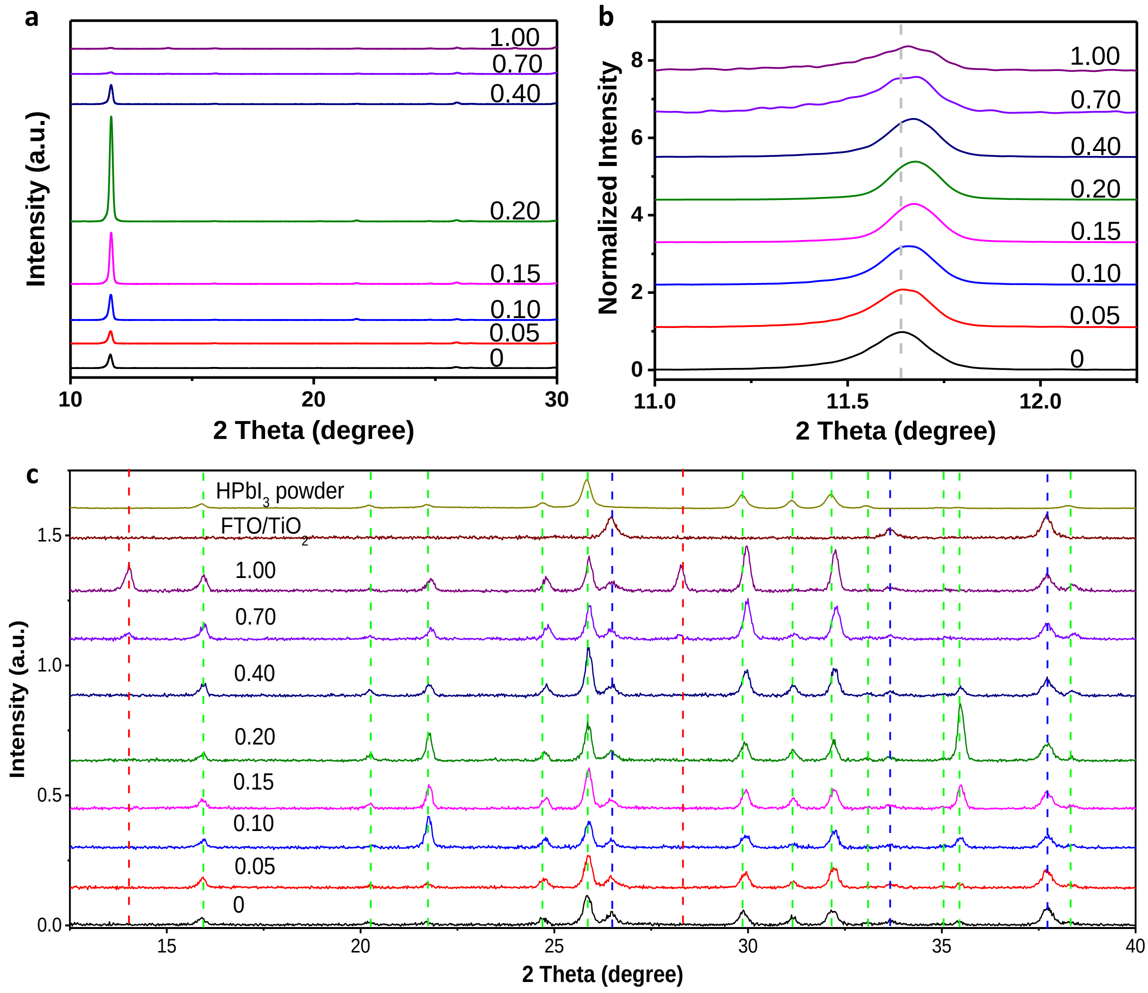

Supplementary Figure 8 │ (a-c) XRD patterns of the $HPbI_3$(Cl) films prepared with different molar ratios of MACl versus $HPbI_3$ in the $HPbI_3$/MACl precursor solution, red dash line (perovskite peaks), green dash line ($HPbI_3$ peaks), blue dash line (FTO/TiO_2 peaks).

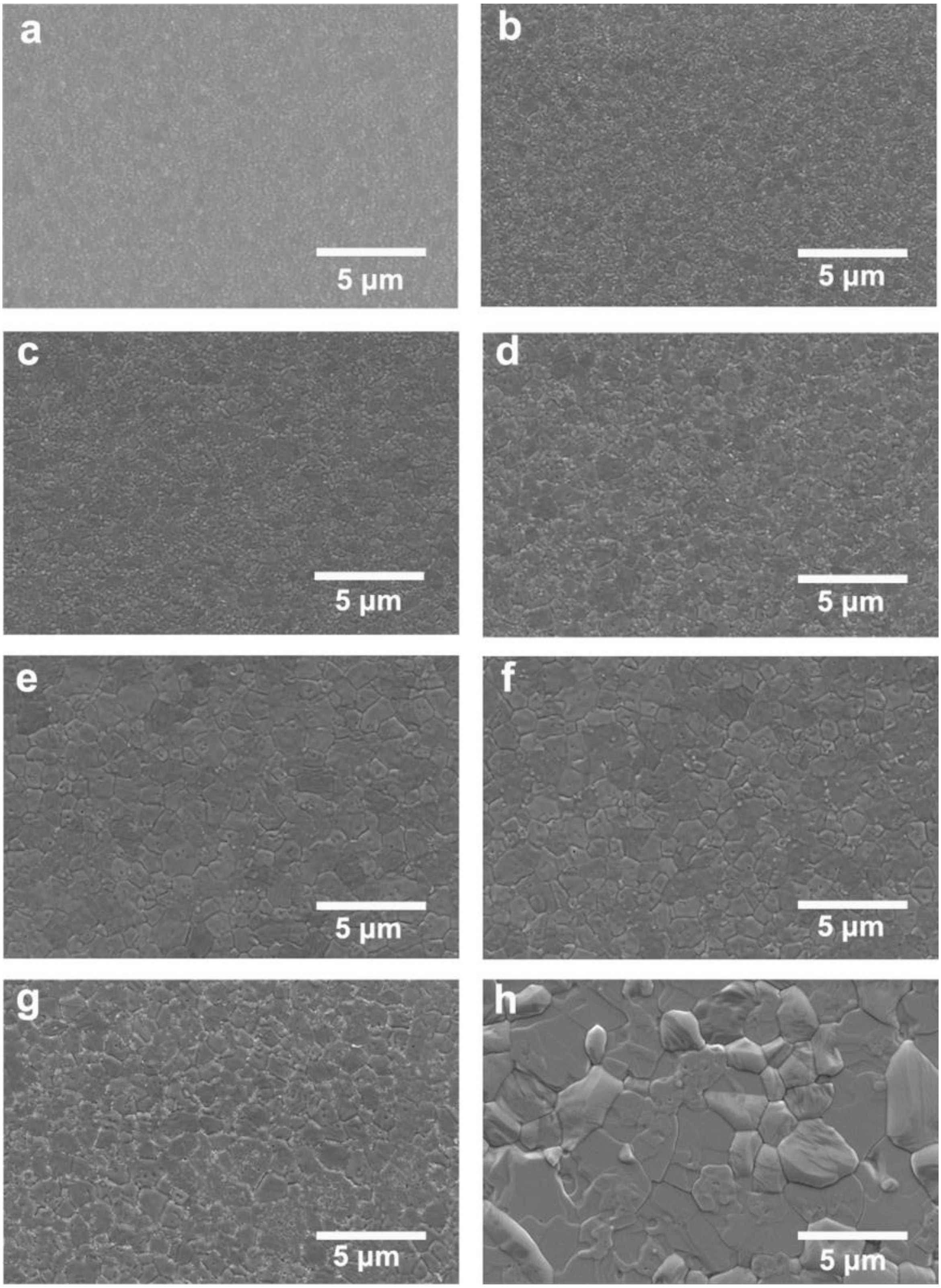

Supplementary Figure 9 │ Top-view SEM images of $MAPbI_3(Cl)$ films prepared with molar ratio of MACl versus $HPbI_3$ of (a) 0, (b) 0.05, (c) 0.10, (d) 0.15, (e) 0.20, (f) 0.40, (g) 0.70, (h) 1.00, respectively. These ratios refer to the ones used in the $HPbI_3$/MACl precursor solution. Scale bars: 5 μm.

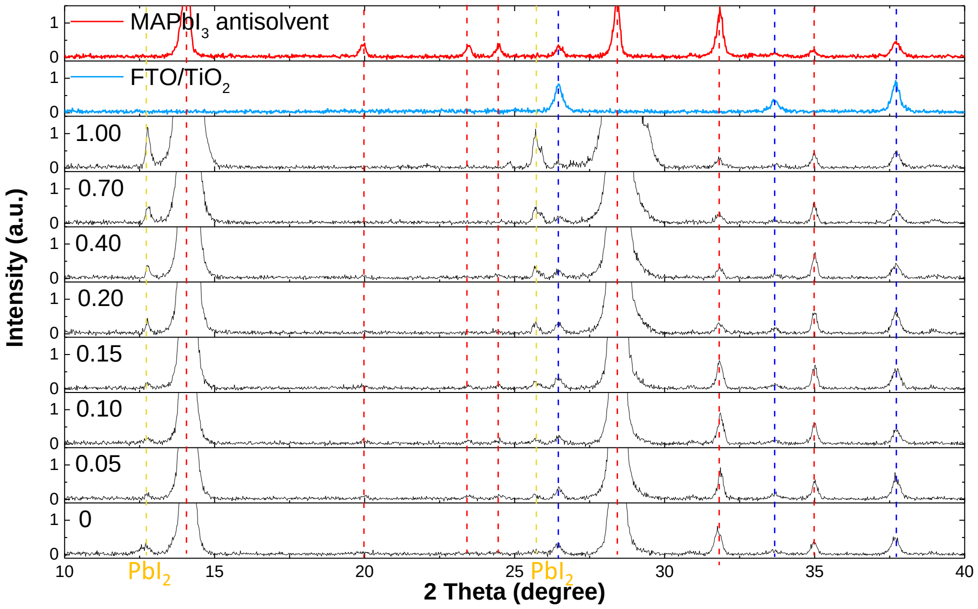

Supplementary Figure 10 │ XRD patterns of $MAPbI_3(Cl)$ films prepared with different molar ratios of MACl versus $HPbI_3$ in $HPbI_3$/MACl precursor solution, $MAPbI_3$ sample prepared by anti-solvent method and FTO/TiO_2 sample, red dash line (perovskite peaks), yellow dash line (PbI_2 peaks), blue dash line (FTO/TiO_2 peaks).

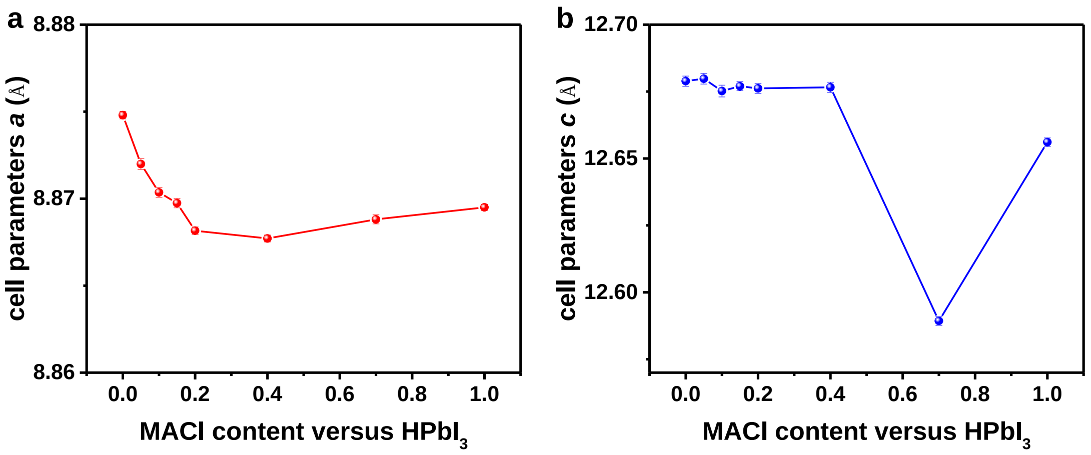

Supplementary Figure 11 │ (a) Tetragonal unit cell parameter *a* and (b) cell parameter *c* for the $MAPbI_3$(Cl) films prepared with molar ratios of MACl versus $HPbI_3$ at, 0, 0.05, 0.10, 0.15, 0.20, 0.40, 0.70, 1.00, respectively, in the $HPbI_3$/MACl precursor solution.

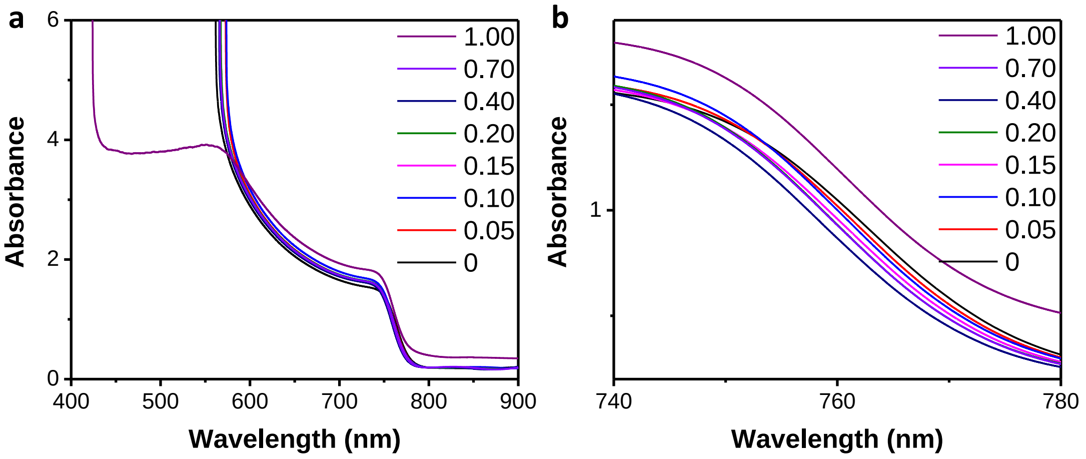

Supplementary Figure 12 | UV-Vis spectra for the $MAPbI_3(Cl)$ films prepared with molar ratios of MACl versus $HPbI_3$ at, 0, 0.05, 0.10, 0.15, 0.20, 0.40, 0.70, 1.00, respectively, in the $HPbI_3$/MACl precursor solution.

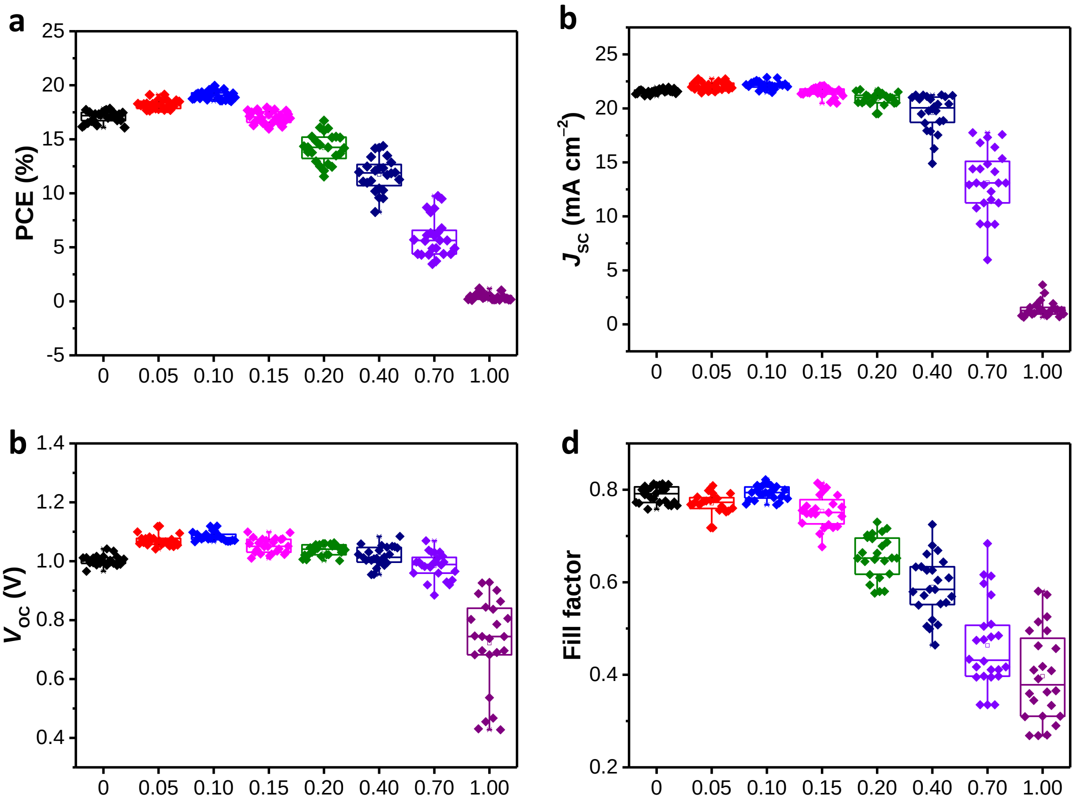

Supplementary Figure 13 | Device performance of the perovskite solar cells based on 1.1 μm thick $MAPbI_3(Cl)$ films prepared with molar ratios of MACl versus $HPbI_3$ at 0, 0.05, 0.10, 0.15, 0.20, 0.40, 0.70, 1.00, respectively, in the $HPbI_3$/MACl precursor solution: (a) PCE, (b) J_{SC}, (c) V_{OC}, (d) fill factor.

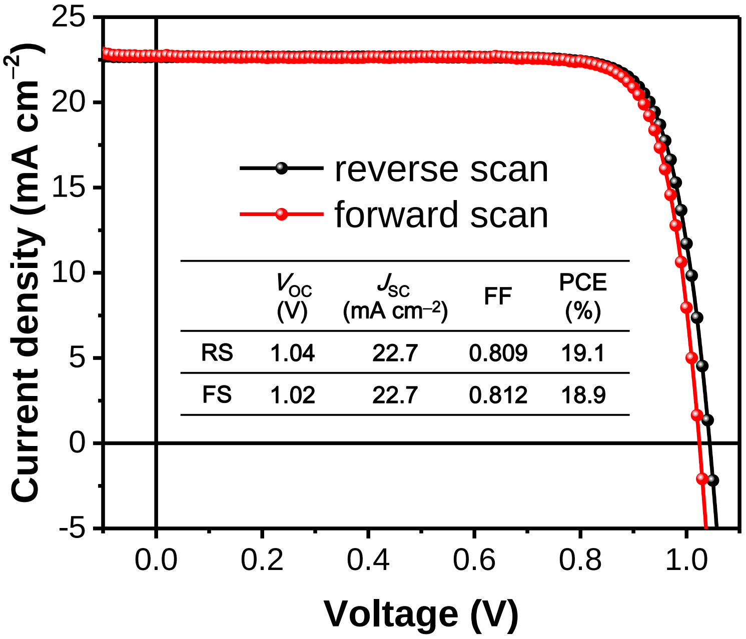

Supplementary Figure 14 | Representative current density-voltage curves of devices based on 1.1 μm thick $MAPbI_3$(Cl) perovskite films using a MACl content of 0.10 in the $HPbI_3$/MACl precursor solution under a scan rate of 25 mV s^{-1}.

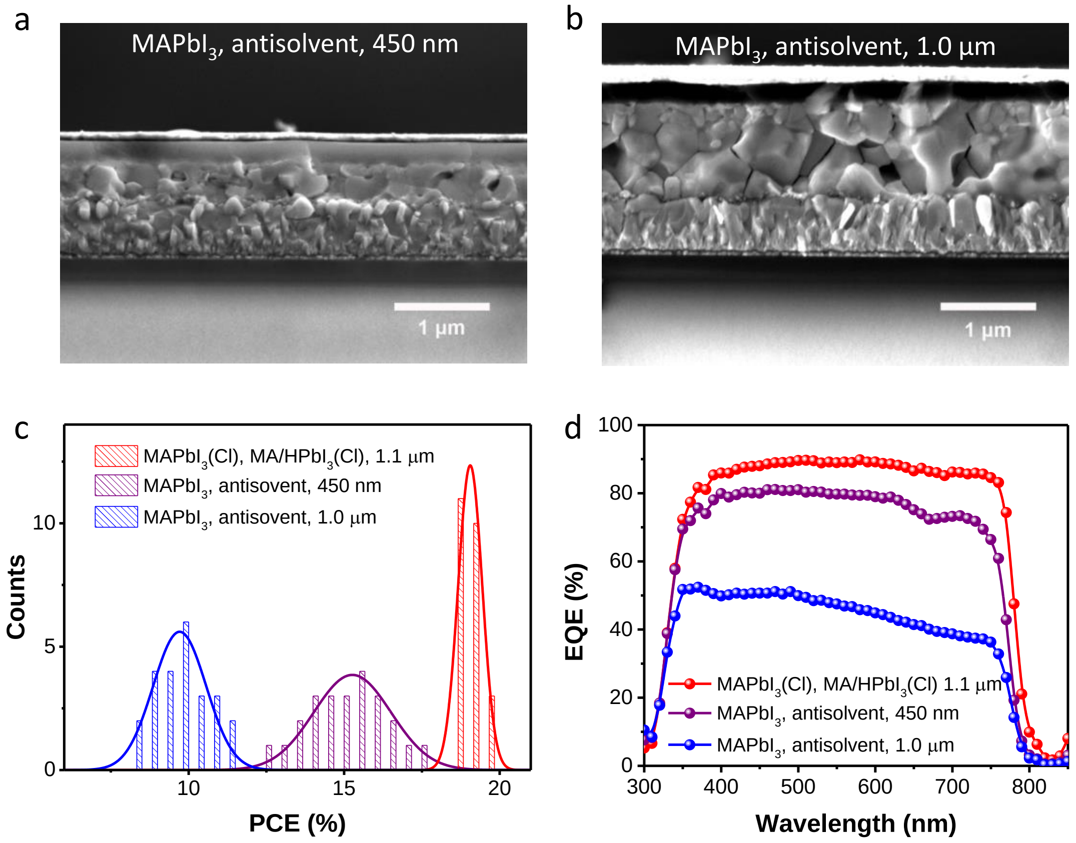

Supplementary Figure 15 │ Cross-sectional-view SEM image of perovskite solar cells based on (a) 450 nm thick and (b) 1.0 μm thick $MAPbI_3$(AS) fabricated by the antisolvent method. (c) Histograms of PCEs and (d) typical EQE spectra for 24 pieces of perovskite solar cells based on the 450 nm thick $MAPbI_3$ films (1.4 M $MAPbI_3$ solution, spin-coating speed, 3000 rpm), 1.0 μm thick $MAPbI_3$ films (2.5 M $MAPbI_3$ solution, spin-coating speed, 2000 rpm) via the antisolvent method, and 1.1 μm thick $MAPbI_3$(Cl) films by the new method developed in this work.

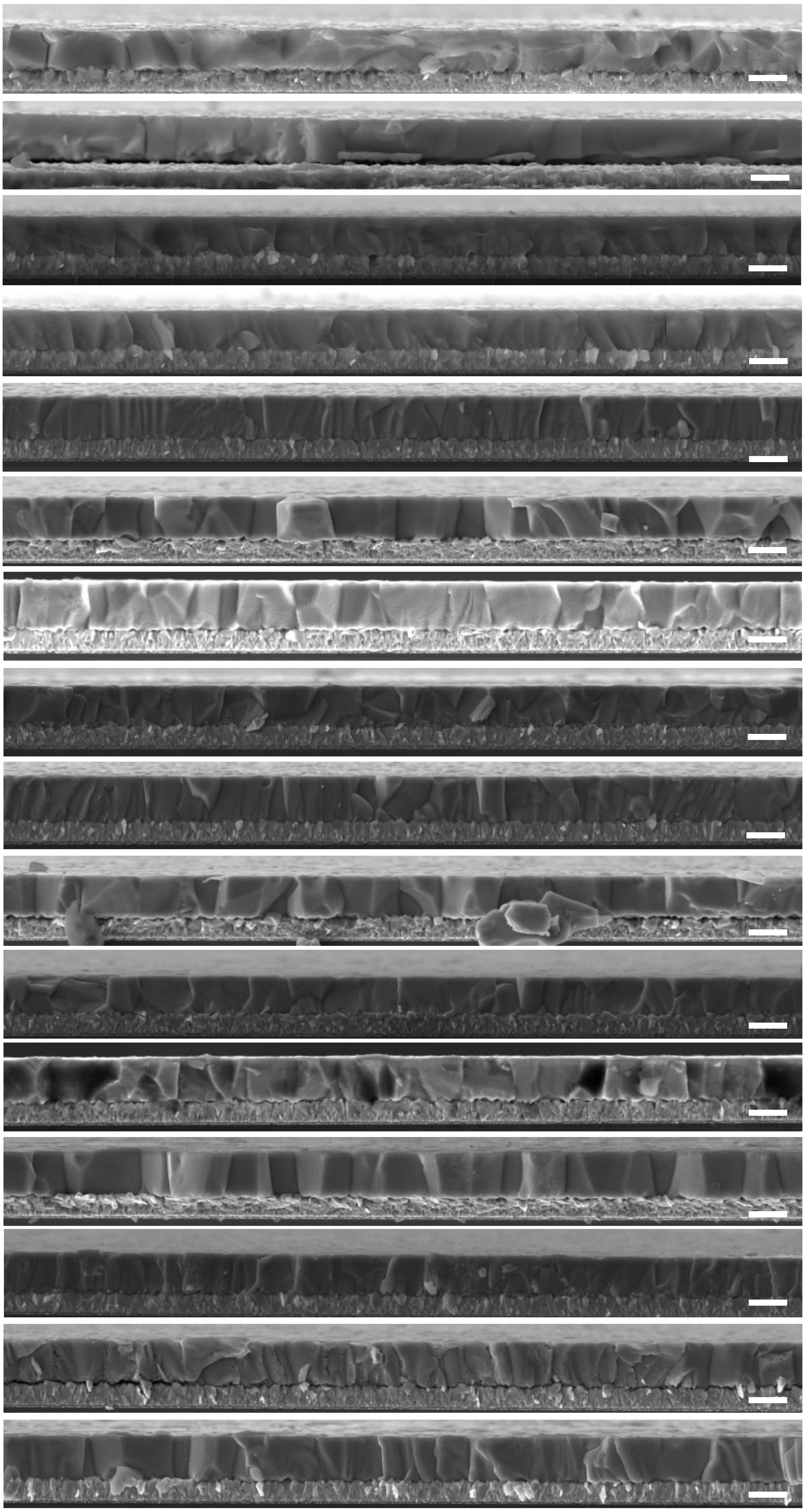

Supplementary Figure 16 | Cross-sectional-view SEM images of 16 $MAPbI_3(Cl)$ samples used for thickness statistics analysis. Scale bars: 1 μm.

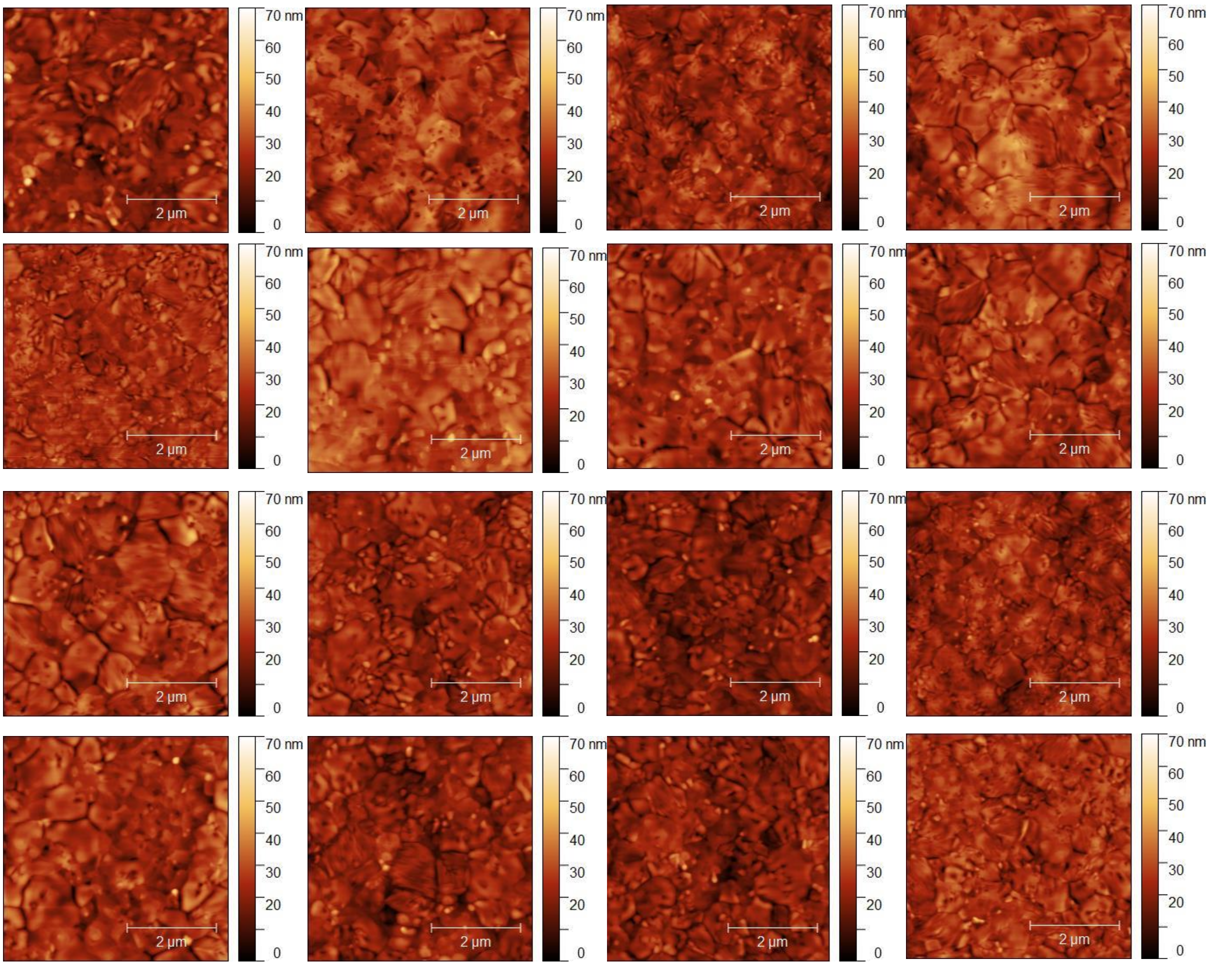

Supplementary Figure 17 | AFM images of 16 $MAPbI_3(Cl)$ samples used for roughness statistics analysis. Scale bars: 2 μm.

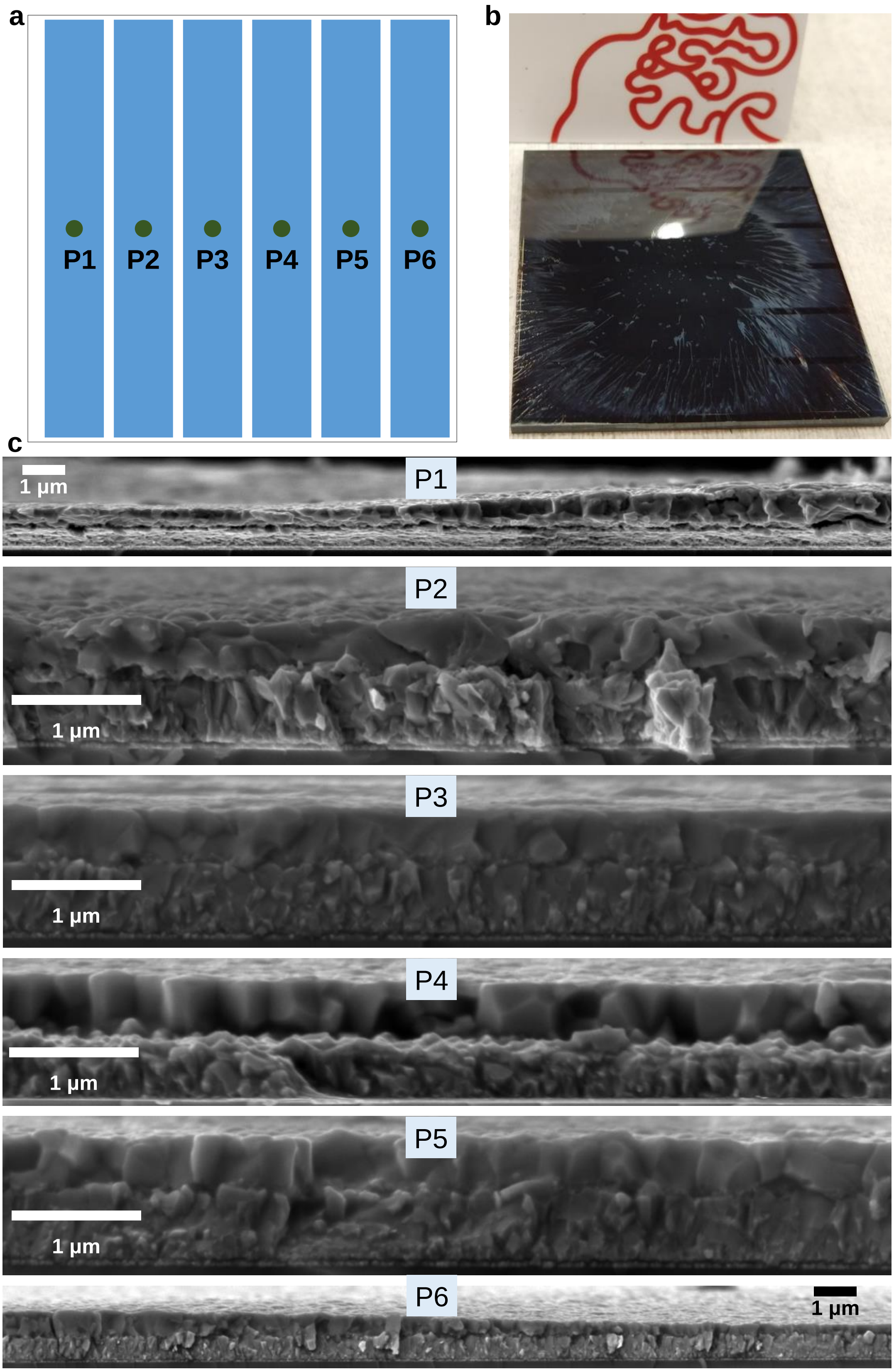

Supplementary Figure 18 | (a) The locations for the film thickness measurement on 5 cm × 5 cm substrates. (b) Photograph of the 450 nm thick $MAPbI_3$(AS) film deposited on a 5 cm × 5 cm substrate with anti-solvent method. (c) Cross-sectional-view SEM images of $MAPbI_3$(AS) film deposited on 5 cm × 5 cm substrates to show the thickness variation.

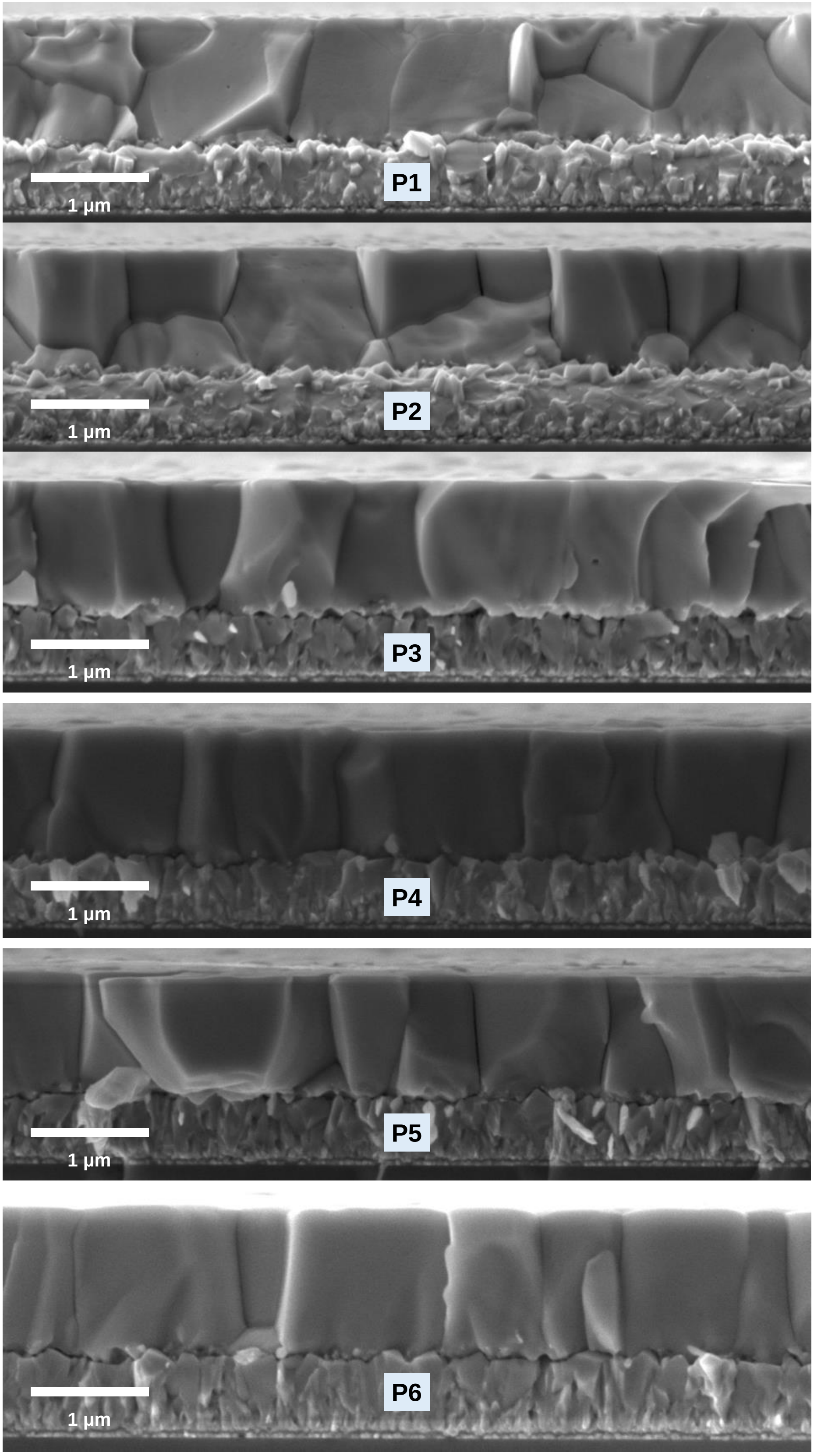

Supplementary Figure 19 │ Cross-sectional-view SEM images of 1.1 μm thick $MAPbI_3(Cl)$ samples on 5 cm × 5 cm substrates used to show the thickness variation.

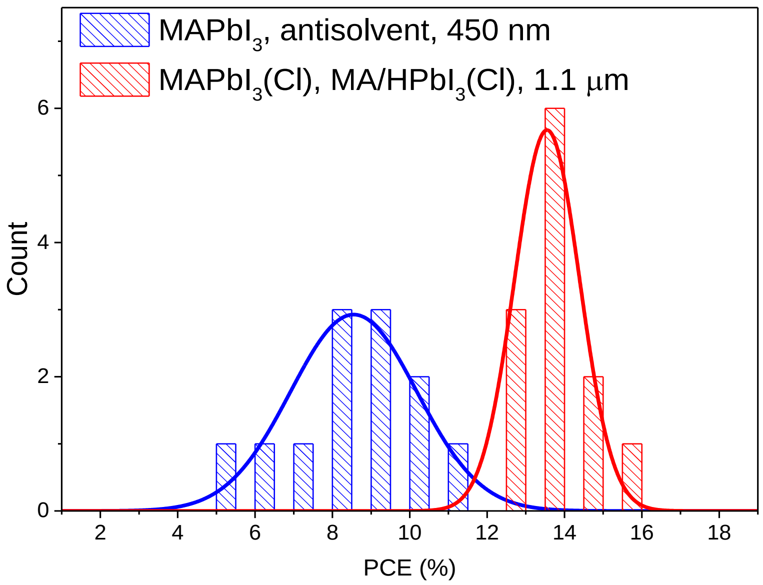

Supplementary Figure 20 | Histograms of PCEs for 12 pieces of perovskite solar modules based on the 450 nm thick $MAPbI_3$(AS) films (1.4 M $MAPbI_3$ solution, spin-coating speed, 3000 rpm) via the antisolvent method, and 1.1 μm thick $MAPbI_3$(Cl) films by the new method developed in this work.

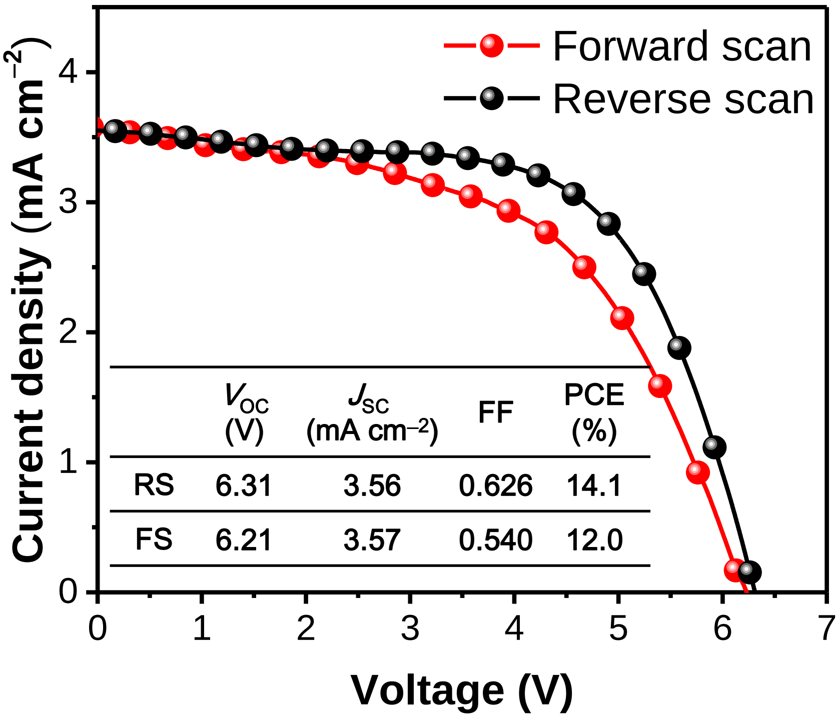

Supplementary Figure 21 | Representative current density-voltage curves of 1.1 μm thick $MAPbI_3$(Cl) films based module devices under reverse scan (RS) and forward scan (FS).

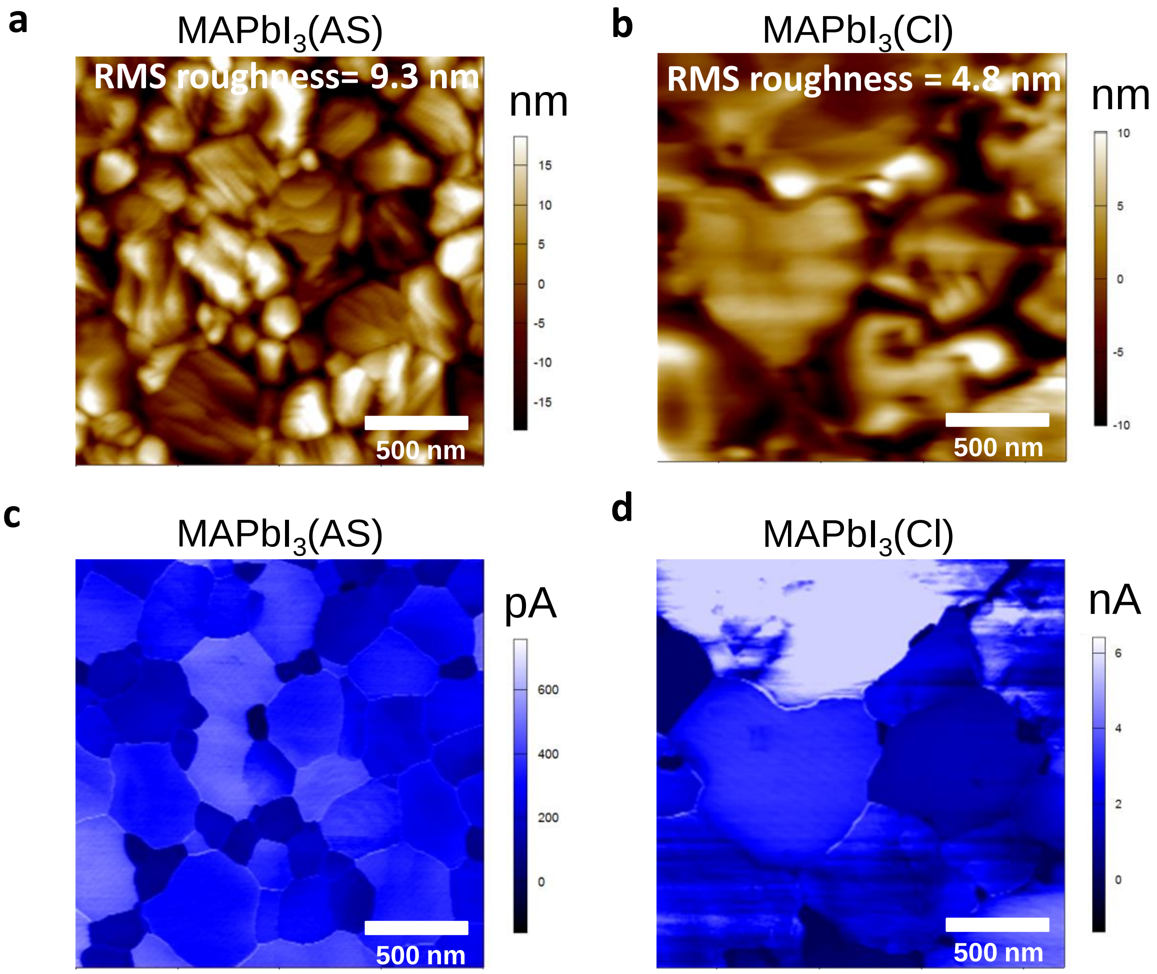

Supplementary Figure 22 | c-AFM images of 1.1 μm-thick $MAPbI_3(Cl)$ film prepared by $HPbI_3(Cl)/CH_3NH_2$ method, and $MAPbI_3(AS)$ film prepared by anti-solvent method.

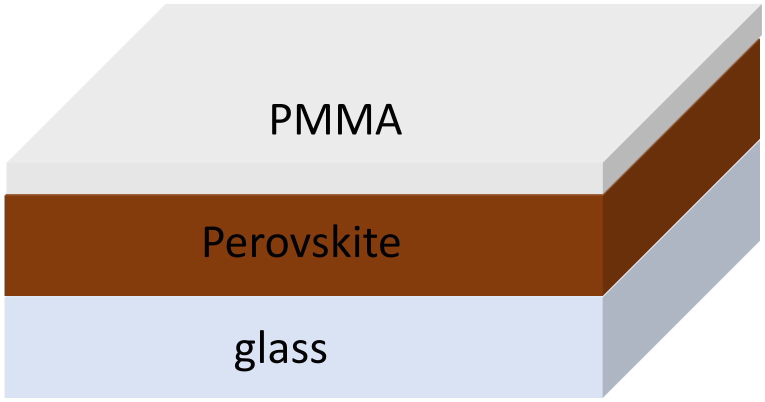

Supplementary Figure 23 | Sample structure for TRPL measurements.

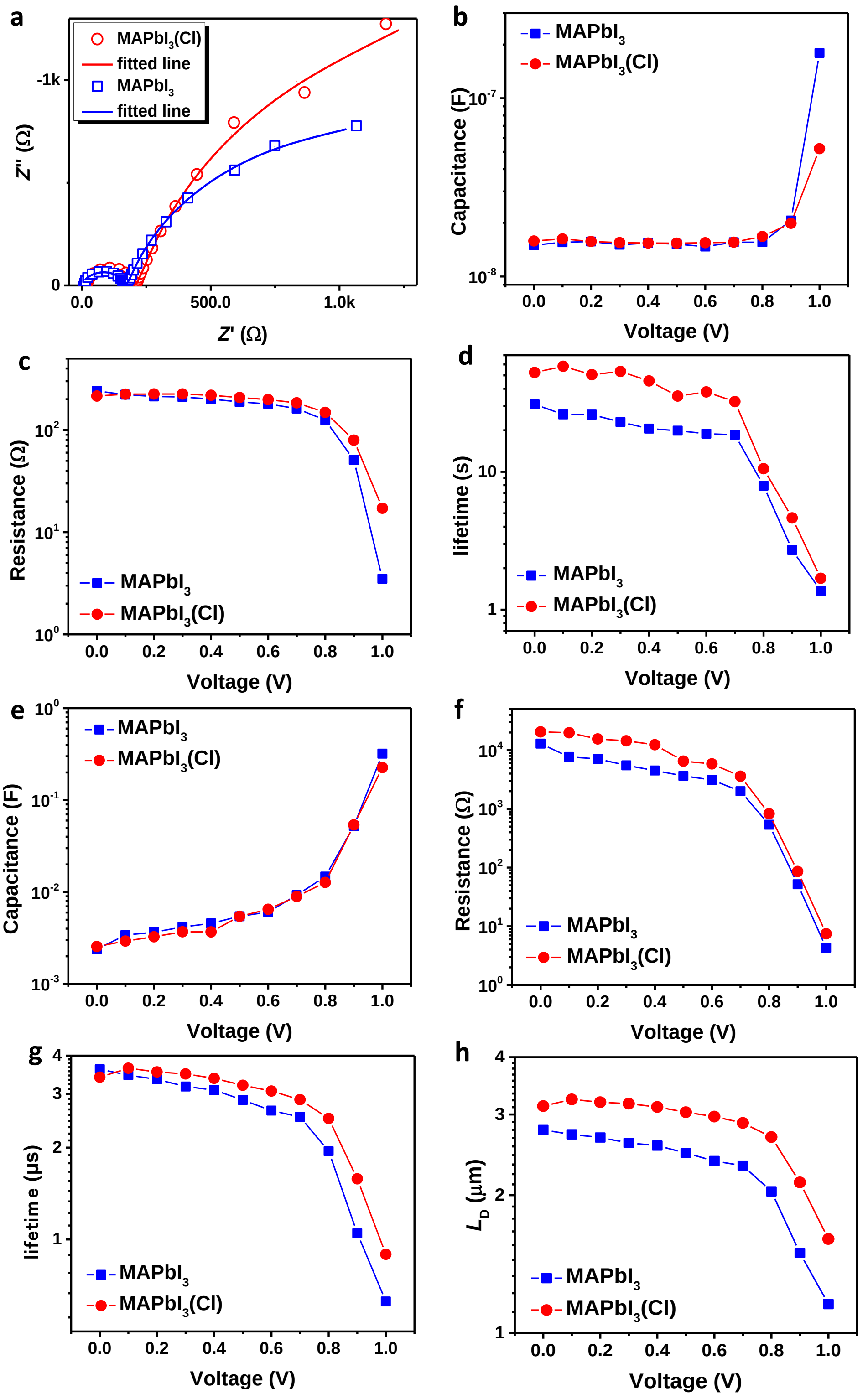

Supplementary Figure 24 | Electrochemical impedance spectroscopy (EIS) characterization. The Nyquist plot (a), low frequency capacitance (b), low frequency resistance (c), low frequency lifetime constant (d), high frequency capacitance (e), high frequency resistance (f) high frequency lifetime constant (g) obtained from the EIS results at different biases for $MAPbI_3$ and $MAPbI_3(Cl)$ device prepared by $HPbI_3(Cl)/CH_3NH_2$ method, respectively. (h) The estimated carrier diffusion length diffusion length for the 1.1 μm thick perovskite films with and without chlorine incorporation prepared by $HPbI_3(Cl)/CH_3NH_2$ method.

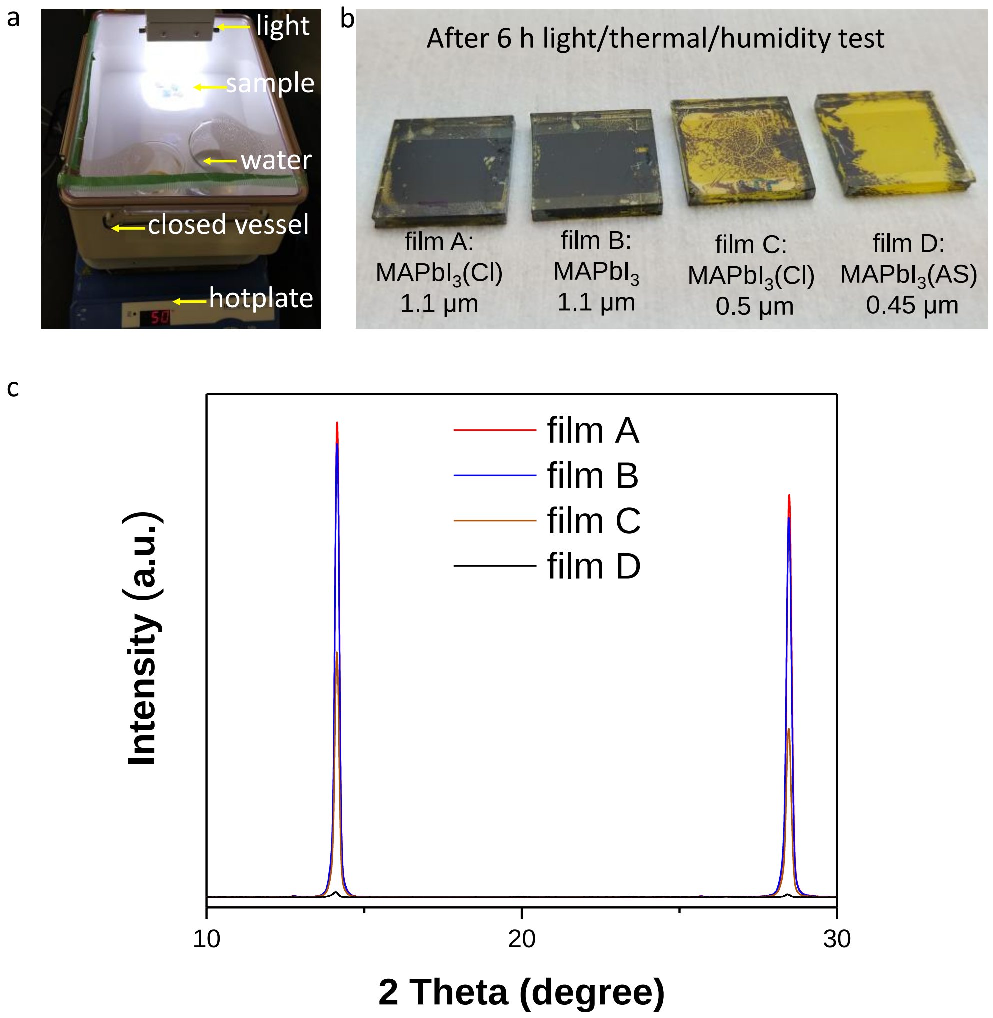

Supplementary Figure 25 │ (a) Home-made stability test apparatus (relative humidity, about 100%; light source, AM1.5G, temperature 50 °C). (b) Optical images of film A: 1.1 μm $MAPbI_3(Cl)$ film prepared by $HPbI_3(Cl)/CH_3NH_2$ method; film B: 1.1 μm $MAPbI_3$ film without substitution of iodine ions by chorine ions prepared by $HPbI_3/CH_3NH_2$ method, film C: 0.5 μm $MAPbI_3(Cl)$ film prepared by $HPbI_3(Cl)/CH_3NH_2$ method; film D: 0.45 μm $MAPbI_3(AS)$ film prepared by anti-solvent method, after 6 h light/thermal/humidity testing. (c) XRD patterns of fresh film A: 1.1 μm $MAPbI_3(Cl)$ film prepared by $HPbI_3(Cl)/CH_3NH_2$ method; film B: 1.1 μm $MAPbI_3$ film without chlorine incorporation prepared by $HPbI_3/CH_3NH_2$ method, film C: 0.5 μm $MAPbI_3(Cl)$ film prepared by $HPbI_3(Cl)/CH_3NH_2$ method; film D: 0.45 μm $MAPbI_3(AS)$ films prepared by anti-solvent method, for comparison of crystallinity.

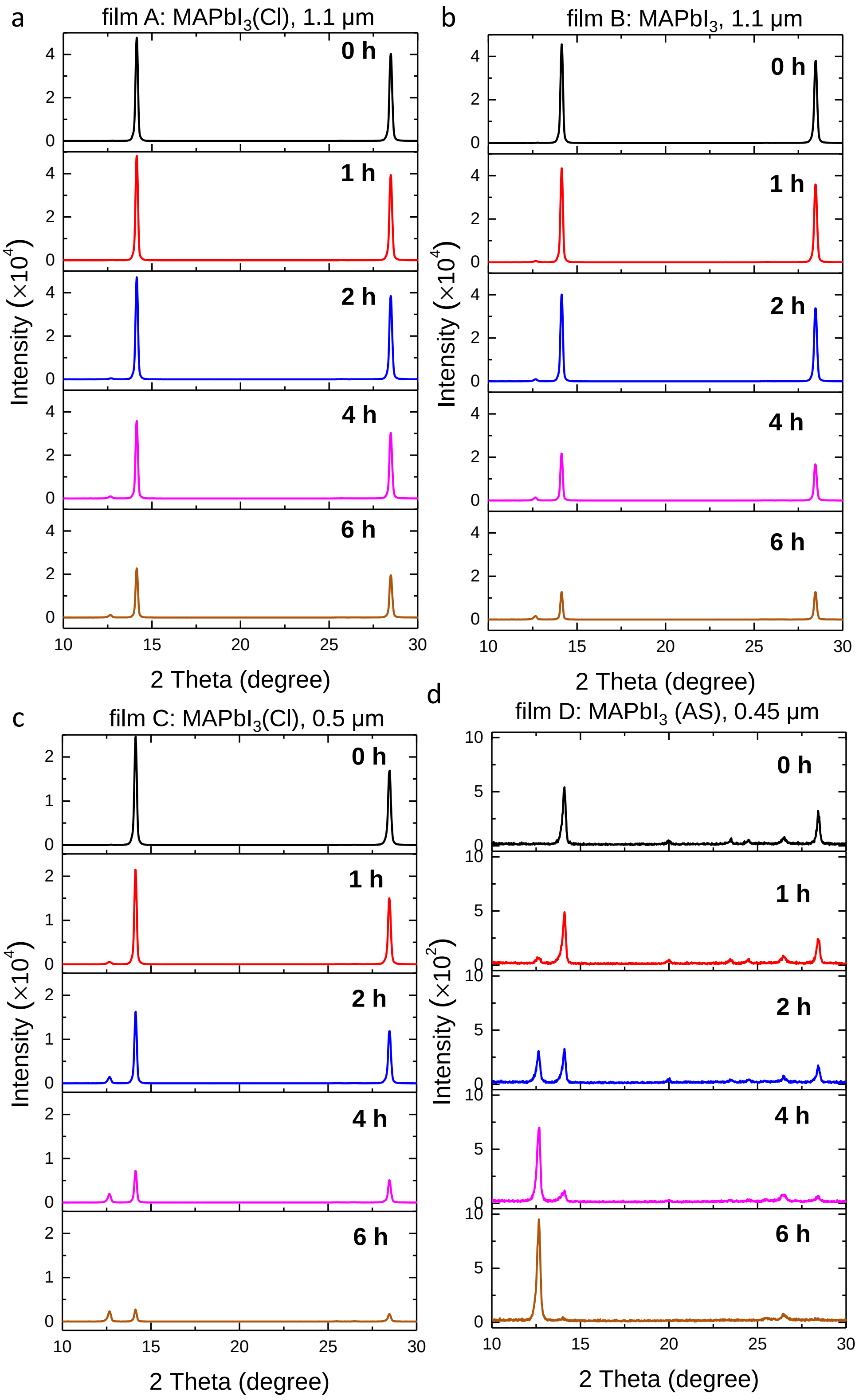

Supplementary Figure 26 | Film stability study of film A: 1.1 μm $MAPbI_3(Cl)$ film prepared by $HPbI_3(Cl)/CH_3NH_2$ method; film B: 1.1 μm $MAPbI_3$ film without substitution of iodine ions by chorine ions prepared by $HPbI_3/CH_3NH_2$ method, film C: 0.5 μm $MAPbI_3(Cl)$ film prepared by $HPbI_3(Cl)/CH_3NH_2$ method; film D: 0.45 μm $MAPbI_3(AS)$ film prepared by anti-solvent method. (**a-d**) XRD patterns of the perovskite films during light/thermal/humidity testing.

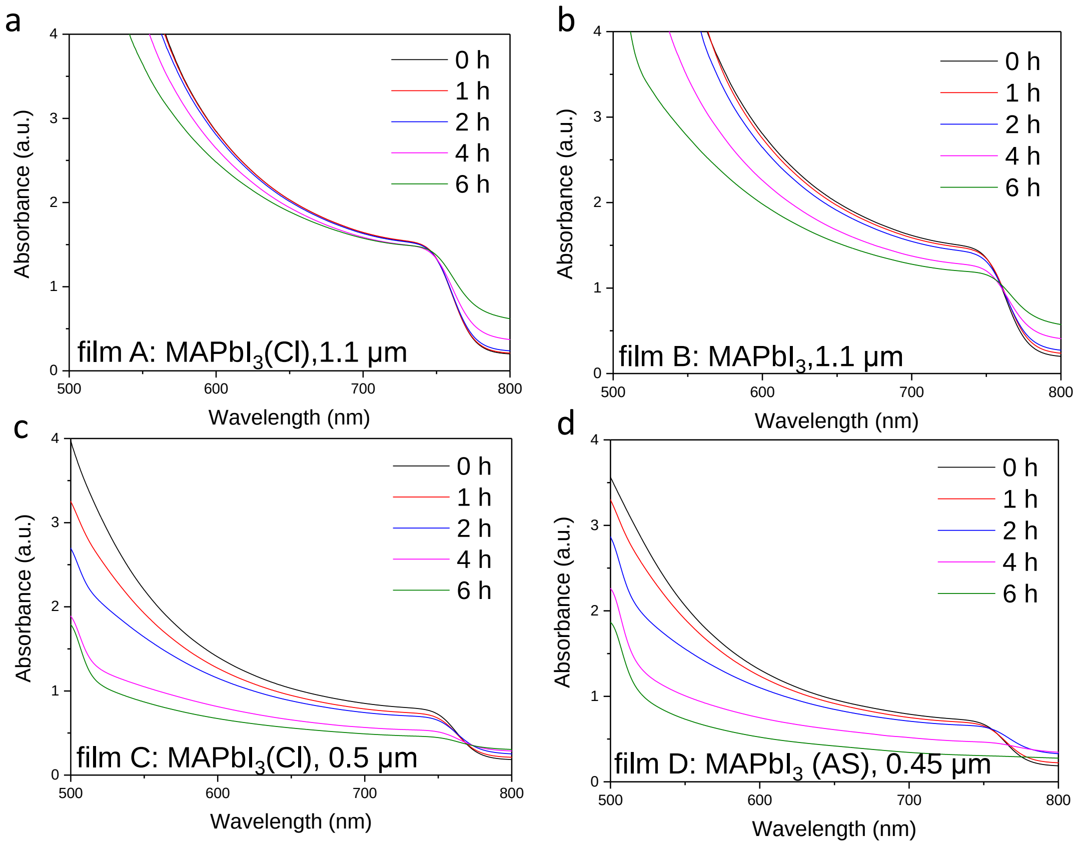

Supplementary Figure 27 | Film stability study of film A: 1.1 μm $MAPbI_3(Cl)$ film prepared by $HPbI_3(Cl)/CH_3NH_2$ method; film B: 1.1 μm $MAPbI_3$ film without substitution of iodine ions by chorine ions prepared by $HPbI_3/CH_3NH_2$ method, film C: 0.5 μm $MAPbI_3(Cl)$ film prepared by $HPbI_3(Cl)/CH_3NH_2$ method; film D: 0.45 μm $MAPbI_3(AS)$ film prepared by anti-solvent method. (**a-d**) UV-vis spectra of the perovskite films during light/thermal/humidity testing.

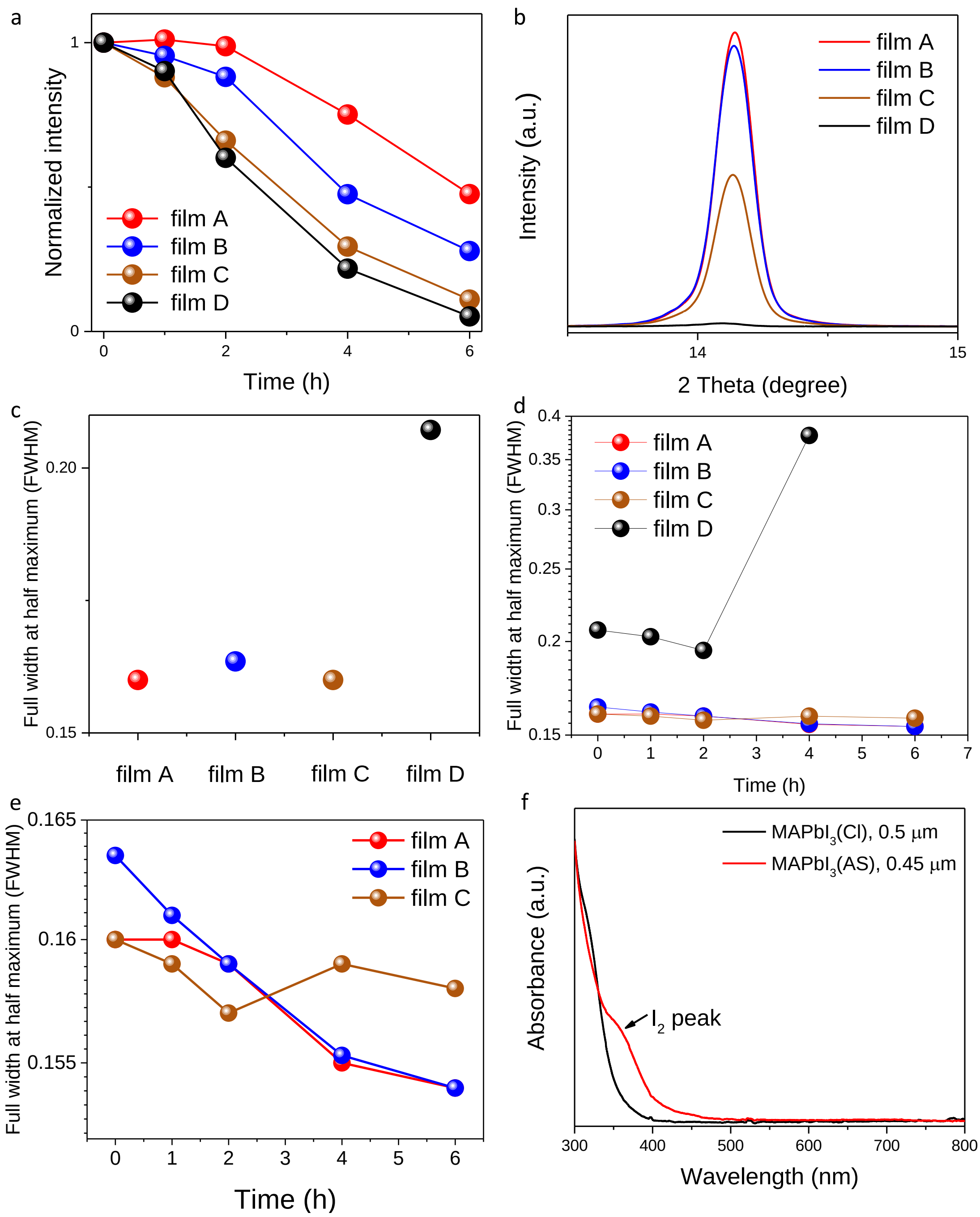

Supplementary Figure 28 | Film stability study of film A: 1.1 μm $MAPbI_3(Cl)$ film prepared by $HPbI_3(Cl)/CH_3NH_2$ method; film B: 1.1 μm $MAPbI_3$ film without substitution of iodine ions by chorine ions prepared by $HPbI_3/CH_3NH_2$ method, film C: 0.5 μm $MAPbI_3(Cl)$ film prepared by $HPbI_3(Cl)/CH_3NH_2$ method; film D: 0.45 μm $MAPbI_3(AS)$ films prepared by anti-solvent method. (**a**) Evolution of XRD peak intensity of perovskite peak around 14.1 ^{o} for four aged films. (**b**) XRD patterns and (**c**) full width at half maximum perovskite peak around 14.1 ^{o} of fresh films for comparison of crystallinity. (**d, e**) Evolution of the full width at half maximum of the perovskite peak at 14.1 ^{o} for the fresh films to compare crystallinity of the four aged films (**f**) UV-vis spectra of the solution prepared samples obtained by dissolving the fresh 0.5 μm $MAPbI_3(Cl)$ film and $MAPbI_3(AS)$ film into H_2O/EtOH (V:V = 1:1) mixture and filtering the suspension through PTFE films.

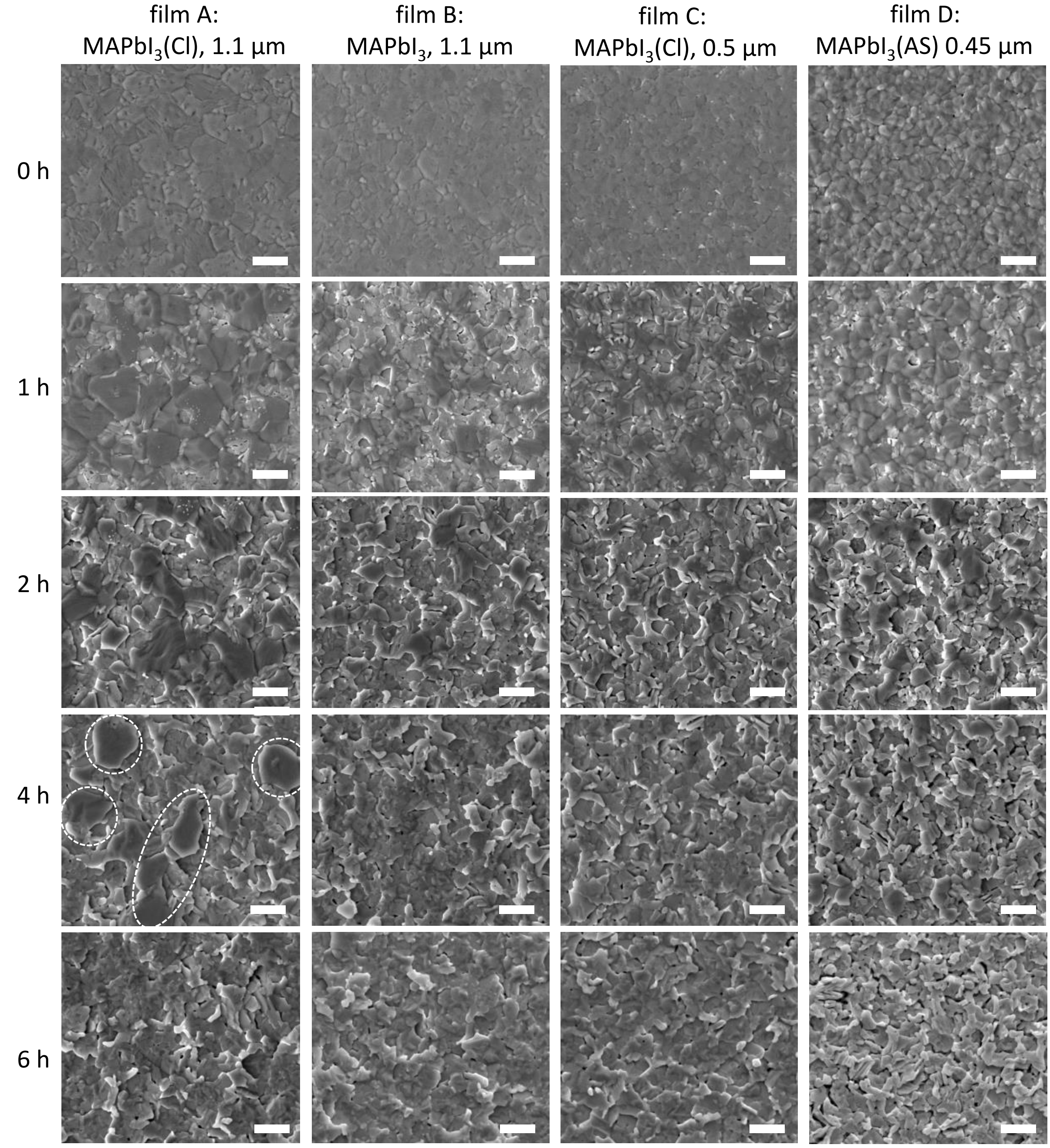

Supplementary Figure 29 │ Top-view SEM images of film A: 1.1 μm $MAPbI_3(Cl)$ film prepared by $HPbI_3(Cl)/CH_3NH_2$ method; film B: 1.1 μm $MAPbI_3$ film without substitution of iodine ions by chorine ions fabricated prepared by $HPbI_3/CH_3NH_2$ method, film C: 0.5 μm $MAPbI_3(Cl)$ film prepared by the $HPbI_3(Cl)/CH_3NH_2$ method; film D: 0.45 μm $MAPbI_3(AS)$ film prepared by anti-solvent method under light/thermal/humidity testing. Scale bar: 1 μm.

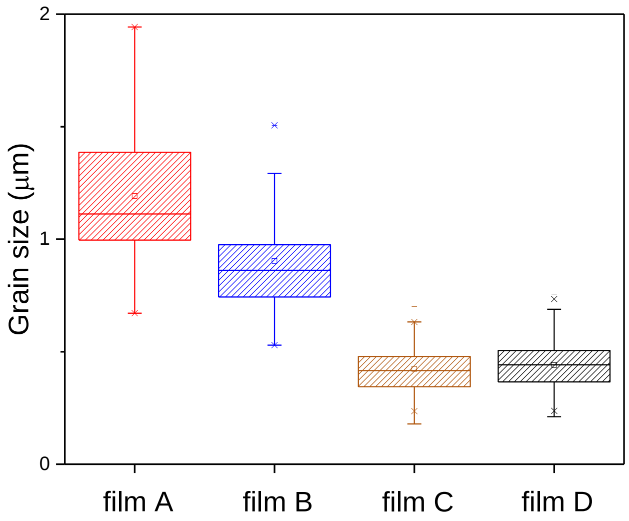

Supplementary Figure 30 | Grain size statistics for film A: 1.1 μm $MAPbI_3(Cl)$ film prepared by $HPbI_3(Cl)/CH_3NH_2$ method; film B: 1.1 μm $MAPbI_3$ film without chlorine incorporation prepared by $HPbI_3/CH_3NH_2$ method, film C: 0.5 μm $MAPbI_3(Cl)$ film prepared by $HPbI_3(Cl)/CH_3NH_2$ method; film D: 0.45 μm $MAPbI_3(AS)$ films prepared by anti-solvent method.

Supplementary Table 1 | Summary of the perovskite film thickness values of perovskite solar cells from representative reports.

Thickness (nm)	PCE (%)	device configuration	references
360	17.5	planar: ITO/c-OTPD/$MAPbI_3$/PCBM/C60/BCP/Al	*Nat. Commun.* **2015**, 6, 7747
400	20.47 (19.6, 1 cm^2)	meso: FTO/TiO_2/$FA_{0.81}MA_{0.15}PbI_{2.51}Br_{0.45}$/spiro-OMeTAD/Au	*Science* **2016**, 354, 206
400	21.02	meso: FTO/TiO_2/$FA_{0.81}MA_{0.15}PbI_{2.51}Br_{0.45}$/spiro-OMeTAD/Au	*Nat. Energy* **2016**, 1, 16142
400	19.3 (12.1, 36 cm^2)	meso: FTO/TiO_2/$MAPbI_3$/spiro-OMeTAD/Au	*Nature* **2017**, 550, 92
500	17.8	planar: ITO/c-OTPD/$MAPbI_3$/PCBM/C60/BCP/Al	*Nat. Commun.* **2015**, 6, 7747
500	19.19	planar: FTO/NiO/$MAPbI_3$/PCBM/Ag	*Adv. Mater.* **2017**, 29, 1701073
500	20.8	meso: FTO/TiO_2/$FA_{0.81}MA_{0.15}PbI_{2.51}Br_{0.45}$(3% excess pbI_2)/spiro-OMeTAD/Au	*Science Advance* **2016**, 2, e1501170
500	21.6	meso: FTO/TiO_2/$Rb_{0.05}(Cs_{0.05}(MA_{0.17}FA_{0.83})_{0.95})_{0.95}Pb(I_{0.83}Br_{0.17})_3$/spiro-OMeTAD/Au	*Science* **2016**, 354, 206
550	18.4	planar: FTO/SnO_2/$MAPbI_{3-x}SCN_x$/spiro-OMeTAD/Au	*Adv. Mater.* **2016**, 28, 5214
600	19.4	planar: ITO/PTAA/$MAPbI_3$/PCBM/C60/BCP/Al	*Nat. Energy* **2016**, 1, 15001
600	20.4	meso: FTO/TiO_2/$MAPbI_3$/spiro-OMeTAD/Au	*Nat. Energy* **2016**, 1, 16081
650	20.0	planar: ITO/SnO_2/$MAPbI_3$/spiro-OMeTAD/Au	*Adv. Mater.* **2017**, 29, 1606774
700	22.1	meso: FTO/TiO_2/$FA_{0.83}MA_{0.17}PbI_{2.49}Br_{0.51}$/spiro-OMeTAD/Au	*Science* **2017**, 356, 1376
770	19.9	planar: ITO/SnO_2/$(FAPbI_3)_{0.97}(MAPbBr_3)_{0.03}$/spiro-OMeTAD/Au	*Nat. Energy* **2017**, 1, 16177

800	16.8	planar: ITO/TiO_2/$MAPbI_3$/spiro-OMeTAD/Au	*ACS Appl. Mater. Interfaces*, **2016**, 8, 34446
820	18.3	FTO/SnO_2/C60-SAM/$MA_{0.7}FA_{0.3}PbI_3$/CZ-TA/Au	*Nano Energy*, **2017**, 40, 163
845	15.1	planar: ITO/PEDOT:PSS/$MAPbI_3$/PCBM/C60/BCP/Al	*Energy Environ. Sci*. **2015**, 8, 1544
900	12.0	planar: ITO/ PEDOT:PSS/doped-polyTPD (0.05% AgSbF6)/$MAPbI_3$/PCBM60/Au	*APL Mater.* **2014**, 2, 081504
1010	16.8	planar: ITO/PEDOT:PSS/$(FASnI_3)_{0.6}(MAPbI_3)_{0.4}$/C60/BCP/Ag	*Nat. Energy* **2017**, 2, 17018
1016	14.8	planar: ITO/PEDOT:PSS/$MAPbI_3$/PCBM/C60/BCP/Al	*Adv. Mater.* **2014**, 26, 6503
1130	20.0 (12 cm^2, 15.3)	meso: FTO/TiO_2/$MAPbI_3$(Cl)/spiro-OMeTAD/Au	***This work***

Supplementary Table 2 | The device performance parameters of perovskite solar cells prepared under different substrate temperatures during the $HPbI_3$ film fabrication step.

substrate temp. (°C)	perovskite thickness	V_{oc} (V)	J_{sc} (mA cm^{-2})	FF	PCE (%)
RT	360-650 nm	0.97±0.01	15.0±1.1	0.710±0.039	10.4±1.2
60	870 nm	0.99±0.02	16.7±1.6	0.752±0.34	12.5±1.7
70	900 nm	0.97±0.02	18.0±0.9	0.779±0.025	13.3±1.0
80	960 nm	0.98±0.01	19.8±0.7	0.769±0.025	14.9±0.8
90	1.1 μm	1.00±0.02	21.6±0.2	0.800±0.017	17.3±0.5
100	1.3 μm	1.00±0.01	19.5±0.9	0.723±0.026	14.2±0.9

Supplementary Table 3 | Comparison of device performance parameters of perovskite solar cells based on the 1.1 μm thick $MAPbI_3(Cl)$ films prepared with different molar ratios of MACl versus $HPbI_3$ at 0, 0.05, 0.10, 0.15, 0.20, 0.40, 0.70, 1.00, respectively, in the $HPbI_3$/MACl precursor solution.

MACl vs. $HPbI_3$ (molar ratio)	V_{oc} (V)	J_{sc} (mA cm^{-2})	FF	PCE (%)
0	1.00±0.02	21.6±0.2	0.7.9±0.018	17.1±0.5
0.05	1.07±0.02	22.1±0.4	0.771±0.022	18.2±0.4
0.10	1.09±0.02	22.2±0.3	0.793±0.015	19.1±0.4
0.15	1.05±0.03	21.5±0.4	0.754±0.035	17.0±0.6
0.20	1.04±0.02	20.9±0.6	0.665±0.046	14.2±1.4
0.40	1.02±0.02	19.6±1.7	0.590±0.064	11.7±1.6
0.70	0.98±0.04	13.1±3.0	0.463±0.095	5.9±1.8
1.00	0.72±0.16	1.4±0.7	0.397±0.097	0.4±0.2

Supplementary Table 4 │ The device performance parameters of perovskite solar cells based on 450 nm thick $MAPbI_3$(AS) films (1.4 M $MAPbI_3$ solution, spin-coating speed, 3000 rpm), 1.0 μm thick $MAPbI_3$(AS) films (2.5 M $MAPbI_3$ solution, spin-coating speed, 2000 rpm) via antisolvent method, and 1.1 μm thick $MAPbI_3$(Cl) films from MA/$HPbI_3$(Cl) methods developed in this work.

	V_{oc} (V)	J_{sc} (mA cm^{-2})	FF	PCE (%)
$MAPbI_3$, antisolvent, 450 nm	1.08±0.02	18.7±1.0	0.759±0.032	15.3±1.2
$MAPbI_3$, antisolvent, 1.0 μm	1.02±0.02	12.9±1.0	0.741±0.040	9.7±0.9
$MAPbI_3$(Cl) this work, 1.1 μm	1.09±0.02	22.2±0.3	0.793±0.015	19.1±0.4

Supplementary Table 5 | The device performance parameters of 12 samples of 5 cm × 5 cm module devices based on the 1.1 μm thick $MAPbI_3(Cl)$ films.

sample	V_{oc} (V)	J_{sc} (mA cm^{-2})	FF	PCE (%)
1	6.65	3.66	0.630	15.3
2	6.31	3.56	0.626	14.1
3	6.46	3.33	0.667	14.3
4	6.39	3.18	0.644	13.1
5	6.46	3.30	0.645	13.7
6	6.44	3.22	0.580	12.0
7	6.36	3.06	0.683	13.3
8	6.48	3.09	0.698	13.9
9	6.37	2.94	0.692	12.9
10	6.35	3.16	0.694	13.9
11	6.38	3.13	0.645	12.9
12	6.44	2.99	0.687	13.2
average	6.42±0.08	3.21±0.21	0.638±0.035	13.6±0.8

Supplementary Table 6 │ The device performance parameters of 12 samples of 5 cm × 5 cm module devices based on 450 nm thick $MAPbI_3$(AS) films (1.4 M $MAPbI_3$ solution, spin-coating speed, 3000 rpm via antisolvent method.

sample	V_{oc} (V)	J_{sc} (mA cm^{-2})	FF	PCE (%)
1	6.09	2.33	0.647	9.2
2	5.87	2.06	0.669	8.1
3	6.25	2.36	0.550	8.1
4	5.64	2.07	0.617	7.2
5	5.86	2.06	0.706	8.5
6	6.14	2.60	0.572	9.1
7	6.25	2.11	0.697	9.2
8	6.56	2.93	0.598	11.2
9	6.42	2.61	0.610	10.2
10	6.00	1.65	0.559	5.6
11	6.05	1.65	0.621	6.2
12	6.24	2.63	0.606	10.0
average	6.11±0.25	2.26±0.40	0.621±0.050	8.6±1.6

Supplementary Note 1.

It is found that the film with a thickness below 700 nm possesses pinholes and large thickness variation, which can be attributed to the large voids between island-like crystals in the raw $HPbI_3$ films and / or less remaining amounts of perovskite on the substrate making it difficult to achieve a full coverage (Supplementary Fig. 4 to 6). As the thickness increases to 870 nm, the perovskite film becomes smoother, but some pinholes are still observable. As shown in Supplementary Table 2, it is found that the device performance parameters, especially short-circuit current density (J_{SC}) and fill factor (FF) gradually increase as a function of the increased thickness and improved coverage when elevating the substrate temperature. This observation suggests that thicker film is not only beneficial in reducing optical loss but also helpful in reducing the risk of direct contact between electron and hole transport materials to suppress charge recombination. It is found that the devices with a perovskite film thickness of approximately 1.1 μm yield the decent device performance, and the especially high J_{SC} of 21.6 mA cm^{-2} can be ascribed to suitable thickness, good optical absorption and full coverage. In the 1.3 μm-thick perovskite film case, precipitates are easy to form on the edge of the substrate when dropping the $HPbI_3$ precursor on the substrate before spin-coating due to higher substrate temperature, leading to the film with a poor coverage at the edge of the substrate. Furthermore, J_{SC} decreases, which is likely a result of inefficient charge collection due to the limited charge diffusion length in this case.

Supplementary Note 2.

The MACl content of 0.10 case delivers the best device performance. As a comparison, the devices based on 1.1 μm thick $MAPbI_3$ without Cl incorporation show average J_{SC} of 21.6 ± 0.2 mA cm^{-2}, V_{OC} of 1.00 ± 0.02 V, FF of 0.79 ± 0.02, and PCE of 17.1 ± 0.5 %. For other substitution of iodine ions by chorine ions content cases, the devices at the MACl content of 0.05 also give an average PCE over 18%, which further supports the effectiveness of partial substitution of iodine ions by chorine ions on device performance enhancement. However, in the cases with a MACl content higher than 0.15, the device performance decrease upon increasing of the MACl content. This result is consistent with the previous studies reporting that excess incorporation of chlorine can deteriorate the optoelectronic properties and lead to lower device performance due to the poor phase stability, non-optimized optoelectronic properties, defects formation or poor morphology.[3,4]

Supplementary Note 3.

1.1 μm thick $MAPbI_3(Cl)$ film shows a surface roughness of 4.8 nm (Supplementary Fig. 22) that is consistent with AFM results (Supplementary Fig. 17). This roughness value is smaller than that of the film prepared by the anti-solvent method (9.3 nm).

Supplementary Note 4.

To better understand charge carrier transport properties of the 1.1 μm thick perovskite films when implemented into PSCs, we carried out electrochemical impedance spectroscopy (EIS) to characterize the device under light illumination. The resistance, capacitance and lifetime constant can be extracted from the EIS data at different biases by fitting the Nyquist plots (Supplementary Fig. 24). It was found that the lifetime at high frequency region (in the range of the microsecond time scale) is related to the carrier lifetime within the devices, which is consistent with other kinds of characterization, such as transient photovoltage decay measurement from previous reports.[3] The device with partial substitution of iodine ions by chorine ions shows larger resistance and longer lifetime than the sample without chlorine. This finding further confirms that the device with partial substitution of iodine ions by chorine ions has a slower carrier recombination rate, which is consistent with TRPL and SCLC measurements. Based on the above carrier behavior results, we could further estimate the carrier diffusion length (L_D) by equation: $L_D = (k_B T \mu \tau_r / e)^{1/2}$, where k_B, T, and e are the Boltzmann constant, absolute temperature, and elementary charge, respectively. It was found that $MAPbI_3(Cl)$ sample shows a long carrier diffusion length over 1.6 μm, which is substantially longer than its thickness and also the carrier diffusion length of non-chlorine sample (Supplementary Fig. 24h).

Supplementary Note 5.

In Supplementary Fig. 28 e, the crystallinity evolution does not have a clear correlation with the stability variation of the films. This is because the full width half maximum of the aged film from the XRD represents the crystallinity of undegraded perovskite crystals within the aged film, which still shows a similar level or slightly higher of crystallinity as the initial films.

Supplementary References

1 Ahn, N. *et al.* Highly reproducible perovskite solar cells with average efficiency of 18.3% and best efficiency of 19.7% fabricated via lewis base adduct of lead (II) iodide. *J. Am. Chem. Soc.* **137**, 8696-8699 (2015).

2 Jiang, Q. *et al.* Enhanced electron extraction using SnO_2 for high-efficiency planar-structure $HC(NH_2)_2PbI_3$-based perovskite solar cells. *Nat. Energy* **1**, 16177 (2016).

3 Chen, Q. *et al.* The optoelectronic role of chlorine in $CH_3NH_3PbI_3(Cl)$-based perovskite solar cells. *Nat. Commun.* **6**, 7269 (2015).

4 Yang, M. *et al.* Perovskite ink with wide processing window for scalable high-efficiency solar cells. *Nat. Energy* **2**, 17038 (2017).
